# Genome Assembly of *Pyrocephalus nanus*: A Step Toward the Genetic Conservation of the Endangered Little Vermilion Flycatcher of the Galapagos Islands

**DOI:** 10.1093/gbe/evae083

**Published:** 2024-04-23

**Authors:** David J Anchundia, Athena W Lam, James B Henderson, Matthew H Van Dam, John P Dumbacher

**Affiliations:** Department of Behavioral and Cognitive Biology, University of Vienna, Vienna 1030, Austria; Charles Darwin Research Station, Charles Darwin Foundation, Santa Cruz, Galapagos, Ecuador; Institute for Biodiversity Sciences and Sustainability, California Academy of Sciences, San Francisco, CA 94118, USA; Institute for Biodiversity Sciences and Sustainability, California Academy of Sciences, San Francisco, CA 94118, USA; Institute for Biodiversity Sciences and Sustainability, California Academy of Sciences, San Francisco, CA 94118, USA; Institute for Biodiversity Sciences and Sustainability, California Academy of Sciences, San Francisco, CA 94118, USA; Institute for Biodiversity Sciences and Sustainability, California Academy of Sciences, San Francisco, CA 94118, USA

**Keywords:** *Pyrocephalus nanus*, Tyrannidae genome, Galapagos, vermilion flycatcher

## Abstract

Incredibly powerful whole genome studies of conservation genetics, evolution, and biogeography become possible for non-model organisms when reference genomes are available. Here, we report the sequence and assembly of the whole genome of the little vermilion flycatcher (*Pyrocephalus nanus*; family Tyrannidae), which is an endemic, endangered, and declining species of the Galapagos Islands. Using PacBio HiFi reads to assemble long contigs and Hi-C reads for scaffolding, we assembled a genome of 1.07 Gb comprising 267 contigs in 152 scaffolds, scaffold N50 74 M, contig N50 17.8 M, with 98.9% assigned to candidate chromosomal sequences and 99.72% of the BUSCO passeriformes 10,844 single-copy orthologs present. In addition, we used the novel HiFiMiTie pipeline to fully assemble and verify all portions of the mitochondrial genome from HiFi reads, obtaining a mitogenome of 17,151 bases, containing 13 protein-coding genes, 22 tRNAs, 2 rRNAs, two control regions, and a unique structure of control region duplication and repeats. These genomes will be a critical tool for much-needed studies of phylogenetics, population genetics, biogeography, and conservation genetics of *Pyrocephalus* and related genera. This genome and other studies that use it will be able to provide recommendations for conservation management, taxonomic improvement, and to understand the evolution and diversification of this genus within the Galapagos Islands.

**Significance**

The genus *Pyrocephalus* (family Tyrannidae) comprises four recognized species with nine subspecies, distributed within North, Central, and South America, including the Galapagos Islands. The taxonomy of the genus is in flux, and the species endemic to Galapagos include one recently extinct species (*Pyrocephalus dubius*), and several declining populations of another vulnerable species (*Pyrocephalus nanus*). This genome will provide valuable reference for much-needed phylogenetic, population genetic, and conservation genetic work on this genus and species. This and additional studies are being done to help advise active management of this species in Galapagos.

## Introduction

The Galapagos Islands are recognized for their geographical isolation, high endemism, and the biogeographic patterns of evolution within the archipelago. Of 213 native bird species recorded, 48 are endemic (Charles Darwin Foundation 2024). Of these endemic species, the complete genome has been assembled at the scaffold level for only four species, including the Medium Ground Finch (*Geospiza fortis*) (Zhang et al. 2014), the Galapagos Flightless Cormorant (*Phalacrocorax harrisi*) (Burga et al. 2017), Galapagos Penguin (*Spheniscus mendiculus*) (Pan et al. 2019), and the Small Tree Finch (*Camarhynchus parvulus*) (Rubin et al. 2022).

Due to increasing anthropogenic pressure and decreasing population sizes of many endemic birds in Galapagos (Fessl et al. 2015; Dvorak et al. 2017, 2020; Geladi et al. 2021), there is a pressing need for high-resolution genetic conservation studies of threatened species in Galapagos. One important endemic species is the Galapagos little vermilion flycatcher, *Pyrocephalus nanus*, which was once distributed throughout most of the Galapagos archipelago (12 islands) (Gifford 1919), but in recent decades, its populations have disappeared from four islands and are on the verge of disappearing from two more (Merlen 2013; Fessl et al. 2015; Leuba et al. 2020).

*Pyrocephalus nanus* is found in multiple distinct climate and ecosystem types (BirdLife International 2023) from xeric/desert ecosystems near the coast (Rothschild and Hartert 1899; Gifford 1919) to the tops of volcanoes with evergreen humid vegetation and frequent precipitation (Mosquera et al. 2022). The lack of a reference genome or detailed genetic study has limited investigations into its population history, taxonomy, evolutionary biology, and its adaptations to various ecosystem types, although preliminary work suggests that there is significant variation among populations (Carmi et al. 2016). Current bird conservation programs in Galapagos aim to recover and restore the former range distribution of this species, and therefore, knowledge obtained from genomic data will be useful for conservation planning and action (García-Dorado and Caballero 2021). The aim of this study is to assemble the whole genome of *P. nanus* as a tool for guiding research and management. Additionally, the genome should be a useful resource for studies of one of the largest avian families Tyrannidae, which includes *Pyrocephalus*.

## Results

### Reference Whole Genome

**Fig. 1.**
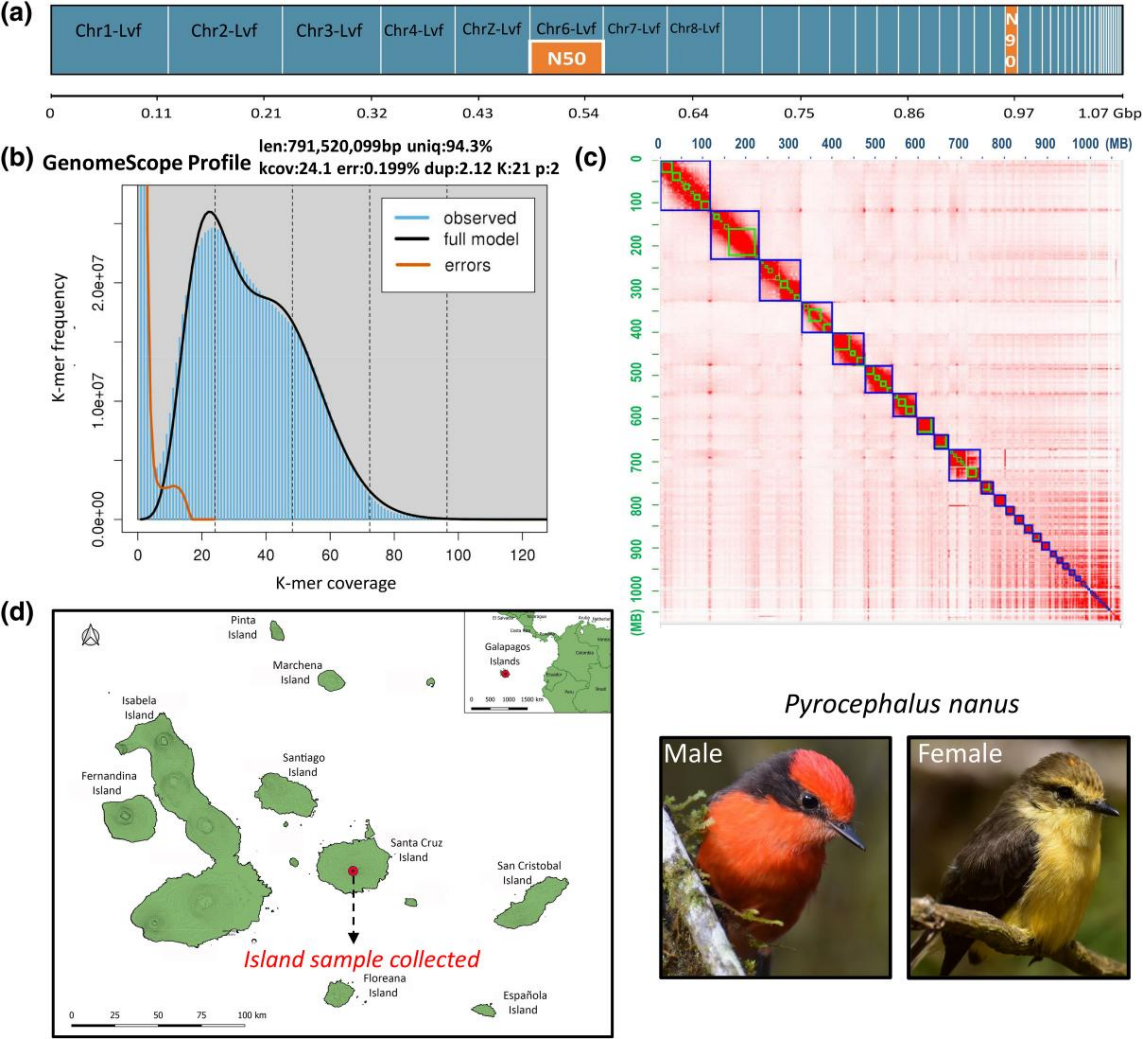
a) Graphical representation of assembled genome statistics, genome size 1.07 Gigabases (Gbp) with N50 and N90 scaffold size comparison graph produced with Quast (Gurevich et al. 2013). Chr 1,2,3… means the chromosome and its number located in the assembled genome of LVF (little vermilion flycatcher), ChrZ-Lvf is the sex chromosome. b) kmer histogram produced with GenomeScope2 to see genome length (len) derived from PacBio sequences and kmer coverage (kcov), read error rate (err), genome unique length (uniq), and duplicate (dup). c) Hi-C contact maps from Juicebox Assembly Tools (JBAT) v.1.11.08 after manual refinement, orientation of contigs, and their delineation within chromosomes, scale in megabases (Mb). d) *Pyrocephalus nanus* is endemic to the Galapagos archipelago, a volcanic island system located in the Eastern Tropical Pacific. A red dot in the center of the map highlights the highlands of Santa Cruz Island *where* the sample was collected. The birds are sexually dimorphic, on the right, you can see the adult plumage.

We sequenced two cells of PacBio HiFi reads that produced 6.84 million reads with 788.79 billion bases, N50 read length 12,741 bp and mean read length 12,551 bp. The Hi-C library produced 103.42 million paired reads, and we retained 99.94 million paired reads after cleaning. We removed 145 sequences of 19,364,419 bases from the HiFiasm contig-only assembly classified by purge_dups as Haplotig, Repeat, Junk, or Highcov. After contig assembly, removal of purge_dup sequences, scaffolding with Hi-C reads, and manual contig placement and orientation, we obtained a final assembly of 1.073 Gb, in 152 scaffolds and 267 contigs (Fig. 1 and Table 1). By comparing the long scaffolds to the chromosome-level assembled genome of the zebra finch (NCBI RefSeq GCF_003957565.2), and using telomere sequences at the end or beginning, we were able to reorganize scaffolds of the *P. nanus* genome into 38 chromosomes (supplementary table S2). Additionally, we were able to identify the fifth largest chromosome as sex chromosome Z (Fig. 1) in addition to the 37 autosomes. Of the 38 chromosome designated sequences, 34 contain at least one telomere and 10 of them have a top and a bottom telomere.

**Table 1:** Sample information assembled genome statistics and accession numbers

Species Scientific name	*Pyrocephalus nanus*	Common name	Little vermilion flycatcher			
Location sample collection	Island: Santa Cruz, Province: Galapagos Islands, Country: Ecuador	Coordinate	S0.63172° W90.36300°			
BioProjects and voucher	NCBI BioProject	PRJNA1040305				
	NCBI BioSample	SAMN38255597				
	NCBI Genome accession	JAWZSU000000000				
	Genome assembly	bPyrNan1_0.fasta				
	NCBI SRA accession raw reads data	PRJNA1040305				
Genome sequence	PacBio HiFi reads	Run	2 PacBio SMRT cells 6.84 million HiFi reads			
	Illumina HiC	Run	NovaSeq 6000 103.42 million pair reads			
Genome assembly quality metrics	Number of scaffolds	152				
	Total size of scaffolds	1,072,479,546				
	Longest scaffold	117,695,848				
	Shortest scaffold	12,661				
	Mean scaffold size	7,055,786				
	Median scaffold size	81,135				
	N50 scaffold length	74,038,366				
	N90 scaffold length	12,777,097				
	L50 scaffold count	6				
	L90 scaffold count	21				
	N50 contig length	17,811,915				
	N90 contig length	4,626,274				
	Number of contigs	267				
	Number of contigs in scaffolds	144				
	Number of contigs not in scaffolds	123				
	GC (%)	42.30				
	Gaps 100 Ns	115				
	BUSCO completeness passeriformes *n* = 10,844	C	S	D	F	M
		99.7%	99.6%	0.1%	0.1%	0.2%
	Organelle	Whole mitochondrial genome 17,151 bp		22 tRNAs, 2 rRNAs, 2 CRs and 13 PCGs		

### Assessment of the Nuclear Genome

GenomeScope2 kmer 21 modeling showed a smaller genome at 791 Mbp than the 1.07 Gbp final assembly size, and 2% heterozygosity with only 45 Mbp repeats. HiFiasm showed kmer peak homozygosity of 24, matching GenomeScope's 24.1 assessment, and did not find a heterozygosity peak. The genome size of 1.07 Gbp had repeats of 120 M bases, 11.20%, which is often underrepresented in the GenomeScope model. To assess the completeness of the genome, we used BUSCO v.5.4.7 (Simão et al. 2015) and compleasm v0.2.2 (Huang and Li 2023) and combined results. BUSCO + compleasm identified Complete BUSCO genes: 99.72% (single-copy orthologs: 99.63%, duplicated: 0.09%, fragmented: 0.06%, missing: 0.22%, *n*: 10,844 genes) (Table 1, see Supplementary material, part II).

### Genome Annotation

Repeatmasker masked 11.20% (see supplementary table S1) of the 1.07 Gbp genome, or 120 Mbp as repeats. This is less than many other avian genomes but similar to several other flycatcher genomes recently sequenced. All annotations were done bioinformatically without RNA-seq data. BRAKER initially found 31,920 start codons and 31,931 stop codons. Functional gene annotation was used to remove low confidence gene models. The final gene annotation included 30,101 genes and 31,748 mRNAs, with a total of 161,303 exons. Genes composed 13.26% of the genome. Gene names and/or descriptions were assigned to 19,303 of the identified mRNAs from the genome. Additional annotation statistics can be found in the supplement, as well as full annotation (.gff, .fna, .faa) files (included as Supplementary material).

### Mitochondrial Genome

A total of 167 HiFiasm corrected HiFi reads mapped to the mitogenome, and the HiFiMiTie pipeline unambiguously assembled these into a complete circular mitochondrial genome of 17,151 nucleotides. The genome has a remnant control region (CR) flanked by tRNA Glu and tRNA Phe, and a primary control region flanked by tRNA Thr and tRNA Pro (see supplementary fig. S1, Supplementary Material online, Supplementary material). A total of 113 HiFi reads contained control region segments: each of the remnant CR sections was 178 nt in length, and each of the primary CR sections was 1,430 nt in length. As typical for avian mitogenomes, there were 22 tRNAs, 2 rRNAs, and 13 protein-coding genes (supplementary fig. S1, Supplementary Material online; see details in part I of Supplementary material).

As a check, the pipeline runs megahit (Li et al. 2016) using the same 167 HiFi reads. The resulting sequence is identical except where the primary control region contains repeats. The issue with repeat contraction or duplication is a common problem with kmer based assemblers such as megahit. The HiFiMiTie primary assembly mode uses segmented multiple sequence alignment and is typically a more reliable tool for resolving repeated regions when highly accurate long reads are available.

## Discussion

Here, we present a highly complete genome of *P. nanus*, and the first genome of a Galapagos passerine assembled with PacBio HiFi sequences. This haploid genome size of 1.073 Gb is similar to other genomes of this bird family that are between 1.0 and 1.1 Gb (Ruegg et al. 2018). Of the eight complete genomes currently assembled on the Tyrannidae family (NCBI), none were made with PacBio HiFi sequences. These other assemblies have a large number of scaffolds ranging from 1,692 to 43,947 with a mean of 16,422 see supplementary table S3, Supplementary Material online, Supplementary material. Using HiFi reads, we were able to obtain longer average contig sizes and with Hi-C reads fewer scaffolds. This high-resolution genome is also an important resource for future studies on the Tyrannidae family (441 species), the largest avian family (Billerman et al. 2020), and the subfamily Fluvicolinae (∼130 species) that includes *Pyrocephalus*, its closest related taxa *Alectrurus*, *Arundicola*, *Gubernetes*, *Colonia*, and several other poorly studied genera (Ohlson et al. 2008; Feng et al. 2020). Also, in our haplotype assembly, we obtained a BUSCO + compleasm complete score of 99.72% suggesting that a great conservation of genes in our assembly and that the genome assembly is highly complete (Huang and Li 2023). This assembly is a step forward to conduct more genomic studies on the endemic species of the Galapagos archipelago.

## Materials and Methods

### Tissue Collection

A single egg of *P*. *nanus* was collected while monitoring nests on Santa Cruz Island during the 2021 breeding season. The nest was blown to the ground by strong winds where the author collected a single broken egg containing a significantly developed but dead embryo. Approximately 40 min after death, the embryo was preserved in 96% alcohol and stored in a freezer at −27 °C. Prior to analysis, the frozen sample was exported from Galapagos to the California Academy of Sciences.

### PacBio High-Molecular-Weight DNA Extraction, Library Preparation, and Sequencing

We prepared libraries using standard PacBio recommendations, but full protocol details for library prep can be found in PacBio (2012) and Van Dam et al. (2021). We confirmed large amounts of high-molecular-weight DNA using FemtoPulse (Agilent, Santa Clara, CA). DNA was sent to the QB3 Genomics facility at the University of California Berkeley for HiFi library preparation and sequencing on a Pacific Biosciences Sequel II platform, sequencing two SMRT cells with HiFi version 2 chemistry.

### In Situ Hi-C Library Preparation

Additional muscle tissues from the same sample were homogenized using a sterile razor blade on ice. An in situ Hi-C library was prepared as described in Rao (2014) with a few modifications. Briefly, after the streptavidin pull-down step, the biotinylated Hi-C products underwent end repair, ligation, and enrichment steps using the NEBNext UltraII DNA Library Preparation kit (New England Biolabs Inc, Ipswich, MA). Titration of the number of PCR cycles was performed as described in Belton (Belton et al. 2012).

### Genome Assembly

For HiFi data preparation, cutadapt v.4.4 (Martin 2011) was used to remove any read with length less than 1000 bp or that contained a PacBio SMRTbell adapter in any position. Cutadapt arguments revcomp, error-rate 0.1, overlap 35, discard, minimum-length 1000 were used along with the –b adapter argument to create cleaned fastq HiFi reads. To assess genome size, we ran jellyfish v2.3.0 (Marçais and Kingsford 2011) using its count option with long reads and kmer size 21, then jellyfish histo for a histogram of kmer frequencies. The histo file was uploaded to GenomeScope2 (qb.cshl.edu/genomescope/genomescope2.0) (Ranallo-Benavidez et al. 2020) to provide estimates of genome properties including total size, repeat content, and heterozygosity.

The two PacBio HiFi cleaned fastq read sets were assembled into genome contigs using the program hifiasm v.0.16.1-r375 (Cheng et al. 2021) and run with arguments —write-ec —write-paf. The HiFiasm program was run via a custom script that converts the program's gfa output to fasta files with any circular records stored separately. Various statistics files were created from the fasta file, including N50, N90, and telomere location in contigs, and BUSCO v.5.4.7 (Simão et al. 2015) was run using the avian passeriformes lineage dataset. To remove haplotypic duplicates, we ran purge_dups v.1.2.5 (Guan et al. 2020) using cutoffs -l 5 -m 36 -u 108 and excluded records from the contig assembly identified as duplicative. To scan for contaminants, we used Kraken2 (Wood et al. 2019) and blastn (Camacho et al. 2009). Taxonomy ID results from the blastn search were translated and sorted by clade, allowing for the identification of any non-avian contigs that were then removed from the contig-level assembly.

To scaffold contigs using Hi-C reads, the Illumina reads from the Hi-C tissue were cleaned and prepared in two steps. First, fastp v.0.23.2 (Chen et al. 2018) was run with the dedup argument to remove Illumina adapters and any read less than 100 bp, and its pair, after adapter removal. Following this, Arima's pipeline (github.com/ArimaGenomics/mapping_pipeline) (Arima 2021) was used with the fastp cleaned Hi-C reads as input to perform additional clean-up and to map the reads to the contig-level assembly with bwa (Li and Durbin 2009; Li 2013). The resulting bam file and the contig assembly were input into the YaHS v.1.2 scaffolding program (Zhou et al. 2023). YaHS scaffolding was run with bam file mapped Hi-C reads and fasta contig assembly input and the –no-contig-ec option. YaHS created .hic and .assembly files that were used to display Hi-C contact maps in Juicebox Assembly Tools (JBAT) v.1.11.08 (Durand et al. 2016a, 2016b) for manual refinement, and we interactively updated the location and orientation of contigs and their delineation within chromosomes.

To assess the level of genome completeness, we ran both compleasm v.0.2.2 (Huang and Li 2023; a reimplementation of BUSCO using miniprot (Li 2023)), and BUSCO v.5.4.7 (Simão et al. 2015) with its default MetaEuk (Levy-Karin et al. 2020) mode, each using the 10,844 ortholog lineage dataset passeriformes_odb10. We updated any BUSCOs not found by compleasm that were found in the BUSCO MetaEuk results.

### Genome Annotation

Prior to gene annotation, regions with repeats were identified using RepeatModeler v2.0.1 (Flynn et al. 2020). We combined repeat models found using RepeatModeler with the avian repeat models from Repbase RepeatMasker libraries v20181026 into a single fasta file. These combined repeat models were used in Repeatmasker v4.0.9 (Smit et al. 2015) with the options -small -xsmall and -nolow to create a soft-masked repeat version of the assembly file used for the gene model structural annotation as well as to create a table of repeat types and lengths.

We annotated the genome without RNA sequence data using BRAKER (version 3.03 April 2023) to predict the location of genes (mRNAs, introns, exons, CDS) using BRAKER's ProtHint pipeline (version 2.6.0 Georgia Tech GeneMark) and AUGUSTUS 3.5.0 (Gabriel et al. 2023) with the vertebrate amino acid sequences from Vertebrata_OrthoDB_10 (Kuznetsov et al. 2023) BRAKER pipeline B., previously called BRAKER2, which is employed when RNA-seq data is unavailable. Potential gene positions were output to gff files. We then eliminated any potential genes that didn’t have both a start and a stop codon and genes that were fully nesting within other genes. Functional annotation of these genes was begun by looking for protein domains in the amino acid sequences found by BRAKER by running InterProScan-5.61-93.0 (with CDD-3.20, FunFam-4.3.0, PANTHER-17.0, Pfam-35.0, PIRSF-3.10, PRINTS-42.0, ProSitePatterns-2022_05, ProSiteProfiles-2022_05, S MART-9.0, SUPERFAMILY-1.75, TIGRFAM-15.0 analyses). The sequences were also blasted against Genbank nt, nr, and swissprot databases downloaded on March 27, 2023 and UNIPROT TrEMBL downloaded on May 15, 2021. Coding sequences were blasted using BLASTN (v2.14) using the nt database. Translated protein sequences were blasted using BLASTP (v2.14) with SwissProt, diamond blastp v.2.1.6 (Buchfink et al. 2021) with the TrEMBL database and with OrthoDB10 vertebrate orthologs. Protein domain IDs and Gene Ontology terms, from InterProScan output, were added to the gff file for each gene and isoform model as were the functional annotation description from the lowest eValue, highest score result from the blast searches. They were also added to the Amino Acid and to the CDS fasta file gene sequences.

### Mitochondrion Assembly

Mitochondrial sequence was derived from corrected HiFi reads from the nuclear genome assembly using an internally created program pipeline named HiFiMiTie (Henderson 2021) version 0.1. The HiFiMiTie pipeline was designed to extract and assemble mitochondrial sequence from PacBio HiFi long reads and also resolve control region heteroplasmy and repeats. It also discovers and annotates tRNAs, rRNAs, protein-coding genes, and up to two duplicate CRs. See details and full logged output in the Supplementary material, part I.

## Supplementary Material

evae083_Supplementary_Data

## Data Availability

The assembled genome is available in NCBI with the reference number submission ID SUB13890500 and the accession number JAWZSU000000000. The raw sequence files used in the assembly are available in the NCBI Sequence Read Archive, accession number PRJNA1040305. Scripts and codes used to perform these analyses are on GitHub (https://github.com/calacademy-research/).
