# Supplementary material for: Genome Assembly of Pyrocephalus nanus: A Step Toward the Genetic Conservation of the Endangered Little Vermilion Flycatcher of the Galapagos Islands
Source: Genome Biol Evol. 2024 Apr 23;16(5):evae083. doi: 10.1093/gbe/evae083 (PMC11077314; doi:10.1093/gbe/evae083)
Supplement: evae083_Supplementary_Data [file evae083_supplementary_data.zip › Supplementary Material_Genome_Pyrocephalus_nanus_3.04.2024DA.docx]

**Supplementary Material**

**Part I. Mitochondrial Genome Assembly using the HiFiMiTie pipeline.**

The HiFiMiTie (hereafter called hfmt) workflow uses known mitochondrial genomes from closely-related taxa (in this case Aves) and blastn (version 2.12.0 with arguments -db mito -query $reads -outfmt "6 std staxid stitle qlen qcovhsp qcovus" -max_target_seqs 5 -taxidlist aves_taxids.txt -evalue 1e-10 -mt_mode 1) to search the taxonomic id matching entries in a copy of the NCBI GenBank mitochondrial database, using HiFi reads as queries. Reads with qcovus 50% or greater are extracted and reverse complemented if necessary to match the subject mitochondrion direction. Candidate reads are annotated with the mitochondrial tRNA finder MiTFi (Allio et al. 2020) using the mito genetic code associated with its taxon, here Aves. Additionally, 12S and 16S rRNAs are discovered using Infernal’s cmsearch. Control region goose hairpins and any OH, OL origins of replication are also annotated. The right neighbor tRNA of tRNAs in each candidate long read are counted and totals used to create the canonical tRNA order of the mitochondrion. Reads with this order for the subset of tRNAs in them are chosen for all downstream sequence creation; though the annotations of all candidate reads are available for additional analysis and corroboration. The full documentation and files are available from github: https://github.com/calacademy-research/HiFiMiTie.

According to convention for vertebrate mitochondrial genomes, the tRNA succeeding the Control Region is chosen as the start of the mitochondrial genome sequence, which is Phenylalanine (tPhe or F) for our genome as well as most vertebrates. This plus the preceding tRNA from the derived canonical order are designated as a first Control Region. Annotated goose hairpins that occur elsewhere in the HiFi reads are used to identify second control regions. When additional goose hairpins are found and one of the control regions is below a threshold size it is identified as a remnant and the other as the primary, which is the case with our data.

After a primary control region is found, three sets of reads are created and each used as input to mafft (Katoh and Standley 2013) to form multiple sequence alignments (msa). The first set is extracted from reads with the tRNA after the CR up to the CR or the end of the read if no CR is after the tRNA. Second set extracted from reads from the beginning of a read up to the tRNA or rRNA that precedes the primary CR. And the third from those reads with both CR flanking tRNAs with the CR in between.

From the mafft msa alignment of the first 2 sets, a consensus sequence is created from each, and the two are combined to create a mitochondrion sequence excluding the CR. The CR reads are analyzed for heteroplasmy, typically different copy numbers of repeats, if any, and representative versions are chosen for additional downstream analysis. The most common CR is used to complete the mitochondrion sequence. The sequence is constructed to start at tRNA tPhe, which canonically begins annotations of vertebrate mitogenomes.

The mitochondrial sequence is annotated by the hfmt pipeline. Separately MITOS2 (Bernt et al. 2013) was used to confirm and check the annotation.

When running HFMT, the program creates an extensive log file that is well annotated for novice users. The complete log file for our mitochondrial assembly is pasted below, and it contains descriptions of the results and output of each step. A settings file records a range of values.

hfmt.log:

[2021-11-15 15:28:43] HiFiMiTie version 0.01 -- Find & Analyze Metazoan Mitochondria from HiFi reads

[2021-11-15 15:28:43] Step 1 -- Setup: taxid and HiFi file(s) to use

[2021-11-15 15:28:44] HiFiMiTie directory hfmt_111521 created

[2021-11-15 15:28:44] HiFi file(s) to search for mitochondrial reads:

[2021-11-15 15:28:44] /home3/jdumbacher/pyrocephalus/asm/hifiasm_tst1/vf_asm.ec.fa

[2021-11-15 15:28:52] Aves mitogenomes, taxid 8782, chosen as search targets. 964 Aves mitogenomes in the /ccg/blastdbs/mito db.

[2021-11-15 15:29:04] Step 2 -- blast_to_mito

[2021-11-15 15:29:04] created hifi_mito_matches to hold the blast output tsv files

[2021-11-15 15:29:07] blastn -db /ccg/blastdbs/mito -query /home3/jdumbacher/pyrocephalus/asm/hifiasm_tst1/vf_asm.ec.fa -outfmt "6 std staxid stitle qlen qcovhsp qcovus" -max_target_seqs 5 -num_threads 32 -taxidlist taxidlist -evalue 1e-10 -mt_mode 1

[2021-11-15 15:43:03] completed in 0h13m56s with completion code 0

[2021-11-15 15:43:03] 1 blasts completed in 0h13m56s

[2021-11-15 15:43:15] top accn info: 2 mito code of NC_007975 183540 | Cnemotriccus fuscatus | cellular organisms; Eukaryota; Opisthokonta; Metazoa; Eumetazoa; Bilateria; Deuterostomia; Chordata; Craniata; Vertebrata; Gnathostomata; Teleostomi; Euteleostomi; Sarcopterygii; Dipnotetrapodomorpha; Tetrapoda; Amniota; Sauropsida; Sauria; Archelosauria; Archosauria; Dinosauria; Saurischia; Theropoda; Coelurosauria; Aves; Neognathae; Passeriformes; Tyrannidae; Cnemotriccus; |

[2021-11-15 15:43:15] mito code chosen: 2

[2021-11-15 15:43:16] Step 2 completed in 0h14m11s

[2021-11-15 15:43:16] Step 3 -- pull_fofn_cand_recs

[2021-11-15 15:43:16] pulling records from /home3/jdumbacher/pyrocephalus/asm/hifiasm_tst1/vf_asm.ec.fa

[2021-11-15 15:46:00] pulled 167 records, excluded 3 with low coverage.

[2021-11-15 15:46:00] created mito_hifi_rec_db to hold a blast database containing mito candidate fasta records

[2021-11-15 15:46:02] Step 3 completed in 0h2m45s

[2021-11-15 15:46:02] Step 4 -- select_mito_features

[2021-11-15 15:46:02] features being retrieved from NC_007975

[2021-11-15 15:46:02] retrieving mito features for NC_007975

[2021-11-15 15:46:02] mito_analyze.py -rec -q NC_007975 -nh | sort -k3,3n | bioawk_cas {print ">" $2"_"$5, $3, $4, fldcat(6,NF); print $1} | fold -w 120

[2021-11-15 15:46:04] top_match_feature_sequences.fasta file created

[2021-11-15 15:46:04] Step 4 completed in 0h0m02s

[2021-11-15 15:46:05] Step 5 -- blast_features

[2021-11-15 15:46:05] created blast_results to hold the blast output tsv files

[2021-11-15 15:46:05] created cr_analysis to hold control region(s) analysis files

[2021-11-15 15:46:05] blastn -db hfmt_111521/mito_hifi_rec_db/mito_hifi_recs -query hfmt_111521/top_match_feature_sequences.fasta -outfmt "6 std staxid stitle qlen qcovhsp qcovus" -num_threads 8 -task blastn -evalue 1e-10 -max_target_seqs 167 | sort -k2,2V -k9,9n -k12,12nr

[2021-11-15 15:46:06] blastn completed in 0h0m01s results in hfmt_111521/blast_results/mito_feature_match_to_cand_recs.tsv

[2021-11-15 15:46:06] blastn -db OH.fas -query hfmt_111521/mito_hifi_recs.fasta -outfmt "6 std staxid stitle qlen qcovhsp qcovus" -max_target_seqs 5 -subject_besthit -evalue .00001 -num_threads 32

[2021-11-15 15:46:09] both feature set and taxname Aves have starting trna of F, setting first trna to F

[2021-11-15 15:46:10] Step 5 completed in 0h0m05s

[2021-11-15 15:46:10] Step 6 -- rna_search

[2021-11-15 15:46:10] cm_results directory created

[2021-11-15 15:46:10] input files checked

[2021-11-15 15:46:10] search for 12S_s-rna

[2021-11-15 15:47:13] search for 16S_l-rna

[2021-11-15 15:51:31] rrna_rrnS.tbl rrna_rrnL.tbl created in cm_results

[2021-11-15 15:51:31] tRNA search mito reads using: MiTFi - mitochondrial tRNA finder

[2021-11-15 16:13:13] mito_hifi_recs.mitfi created in cm_results

[2021-11-15 16:13:14] trna_right_neighbor.matrix created using [1;34mF[0m as the first tRNA.

[2021-11-15 16:13:14] trna order: F V L2 I Q M W A N C Y S2 D K G R H S1 L1 T P E

[2021-11-15 16:13:14] mito_hifi_recs.cm_anno created with mitfi, goose_hairpin, 12S_rna and 16S_rna cm results

[2021-11-15 16:13:14] cm_anno_right_neighbor.matrix created using [1;34mF[0m as the first tRNA.

[2021-11-15 16:13:14] cm_anno rna order: F 12S V 16S L2 I Q M W A N C Y S2 D K G R H S1 L1 T gh P E

[2021-11-15 16:13:15] one_line_per_rec.cm_anno.srt created

[2021-11-15 16:13:15] Step 6 completed in 0h27m05s

[2021-11-15 16:13:15] Step 7 -- CR_analysis

[2021-11-15 16:13:16] grep -e trnE -e trnF -e ^Glu -e ^Phe hfmt_111521/blast_results/mito_feature_match_to_cand_recs.tsv

[2021-11-15 16:13:16] trnE_trnF_distances.tsv created

[2021-11-15 16:13:17] ControlRegion_btw_trnE_trnF_length.stats created

[2021-11-15 16:13:18] CR1 has type remnant, with mean length 178 bp found from 113 reads containing it. flanking trnas E and F

ControlRegion_btw_trnE_trnF_length.stats

Num CR_len

113 178

recs: 113

mean: 178

stddev: 0

mode: 178

longest: 178

shortest: 178

diff: 0

num within one stddev: 113 100.00%

[2021-11-15 16:13:18] grep -e trnT -e trnP -e ^Thr -e ^Pro hfmt_111521/blast_results/mito_feature_match_to_cand_recs.tsv

[2021-11-15 16:13:18] trnT_trnP_distances.tsv created

[2021-11-15 16:13:19] ControlRegion_btw_trnT_trnP_length.stats created

[2021-11-15 16:13:20] CR2 has type Control Region, with mean length 1430 bp found from 99 reads containing it. flanking trnas T and P

ControlRegion_btw_trnT_trnP_length.stats

Num CR_len

99 1430

recs: 99

mean: 1430

stddev: 0

mode: 1430

longest: 1430

shortest: 1430

diff: 0

num within one stddev: 99 100.00%

[2021-11-15 16:13:20] Step 7 completed in 0h0m05s

[2021-11-15 16:13:20] Step 8 -- split_recs_into_sets

[2021-11-15 16:13:20] created split_sequences to hold sequences for assembly by alignment

[2021-11-15 16:13:21] tRNAs T P flank the Control Region.

[2021-11-15 16:13:21] Splitting sequences from Pro to sequence end.

[2021-11-15 16:13:21] Splitting sequences from sequence beginning to Thr.

[2021-11-15 16:13:21] Splitting sequences from Thr to Pro to capture the Control Region and its flanks

[2021-11-15 16:13:21] Split sequences created for alignment assembly and for Control Region assembly & repeat analysis

[2021-11-15 16:13:21] Step 8 completed in 0h0m01s

[2021-11-15 16:13:21] Step 9 -- assemble_mito

[2021-11-15 16:13:22] Step 9a -- assemble using megahit

[2021-11-15 16:13:22] running megahit to assemble mito records using sequences in mito_hifi_recs.fasta

[2021-11-15 16:13:24] megahit_best.fa mito file created

[2021-11-15 16:13:24] running MiTFi to determine location of Phe so that the sequence will start there

[2021-11-15 16:17:06] MiTFi run on megahit_best.fa completed

[2021-11-15 16:17:06] mito_megahit.fasta reoriented to start with Phe.

[2021-11-15 16:17:06] Running MiTFi on the reoriented sequence.

[2021-11-15 16:20:49] input files checked

[2021-11-15 16:20:49] search for 12S_s-rna

[2021-11-15 16:20:50] search for 16S_l-rna

[2021-11-15 16:20:52] mito_megahit_rrnS.tbl mito_megahit_rrnL.tbl created in megahit_out for mito_megahit.fasta

[2021-11-15 16:20:52] goose hairpin sequence information added, if any found

[2021-11-15 16:20:52] mito_megahit.cm_anno with rrns, rrnL, cr and any goose hairpins added to mitfi results created for mito_megahit.fasta

[2021-11-15 16:20:52] MiTFi annotation of mito_megahit.fasta completed, results in mito_megahit.cm_anno

[2021-11-15 16:20:52] Step 9b -- assemble using multi-sequence alignment (msa) consensus

[2021-11-15 16:20:52] running mafft --auto Pro_to_end.fasta >Pro_to_end.mafft.fa 2>Pro_to_end.mafft.log

[2021-11-15 16:22:11] consensus_from_fasta_alignment.sh Pro_to_end.mafft.fa >Pro_to_end.consensus.fa

[2021-11-15 16:22:11] running multiple sequence alignment on reversed sequences of beg_to_Thr.fasta

[2021-11-15 16:22:11] running mafft --auto reversed_beg_to_Thr.fa >reversed_beg_to_Thr.mafft.fa 2>reversed_beg_to_Thr.mafft.log

[2021-11-15 16:23:32] consensus_from_fasta_alignment.sh reversed_beg_to_Thr.mafft.fa >reversed_beg_to_Thr.consensus.fa

[2021-11-15 16:23:32] re-reversing consensus file to make forward version beg_to_Thr.consensus.fa

[2021-11-15 16:23:32] running mafft --auto Thr_CR_Pro.fasta >Thr_CR_Pro.mafft.fa 2>Thr_CR_Pro.mafft.log

[2021-11-15 16:25:07] consensus_from_fasta_alignment.sh Thr_CR_Pro.mafft.fa >Thr_CR_Pro.consensus.fa

[2021-11-15 16:25:07] non_cr_consensus.fasta from Pro_to_end.consensus.fa & beg_to_Thr.consensus.fa

[2021-11-15 16:25:07] running mafft --localpair <(cat Pro_to_end.consensus.fa beg_to_Thr.consensus.fa) >non_cr_consensus.mafft.fa 2>non_cr_consensus.mafft.log

[2021-11-15 16:25:15] consensus_from_fasta_alignment.sh non_cr_consensus.mafft.fa >non_cr_consensus.fasta

[2021-11-15 16:25:15] running mitfi -code 2 analysis on non_cr_consensus.fasta

[2021-11-15 16:28:57] non_cr_consensus.mitfi created with 22 entries

[2021-11-15 16:28:57] removing 1374 from beginning and removing 0 from the end to create 15721bp mito_msa_no_cr.fasta

[2021-11-15 16:28:57] mito_msa_no_cr.fasta created

[2021-11-15 16:28:57] running mitfi -code 2 analysis on mito_msa_no_cr.fasta

[2021-11-15 16:32:23] mito_msa_no_cr.mitfi created with 22 entries

[2021-11-15 16:32:23] input files checked

[2021-11-15 16:32:23] search for 12S_s-rna

[2021-11-15 16:32:24] search for 16S_l-rna

[2021-11-15 16:32:37] mito_msa_no_cr_rrnS.tbl mito_msa_no_cr_rrnL.tbl created in hfmt_111521/msa_assembly/cm_mitfi for mito_msa_no_cr.fasta

[2021-11-15 16:32:37] mito_msa_no_cr.cm_anno created with rrnS, rrnL, cr and any goose hairpin added to mitfi results

[2021-11-15 16:32:38] running mitfi -code 2 -onlycutoff analysis on Thr_CR_Pro.consensus.fa

[2021-11-15 16:33:01] Thr_CR_Pro.consensus.mitfi created with 2 entries

[2021-11-15 16:33:02] mito_msa.fasta created

[2021-11-15 16:33:02] Control Region followed by Pro (P) but first_trna set to Phe, file will be reflowed to begin with Phe (F)

[2021-11-15 16:33:02] mito_msa.fasta reflowed to begin with Phe (F)

[2021-11-15 16:33:02] running mitfi -code 2 analysis on mito_msa.fasta

[2021-11-15 16:36:44] mito_msa.mitfi created with 22 entries

[2021-11-15 16:36:45] input files checked

[2021-11-15 16:36:45] search for 12S_s-rna

[2021-11-15 16:36:46] search for 16S_l-rna

[2021-11-15 16:36:48] mito_msa_rrnS.tbl mito_msa_rrnL.tbl created in hfmt_111521/msa_assembly/cm_mitfi for mito_msa.fasta

[2021-11-15 16:36:48] mito_msa.cm_anno created with rrnS, rrnL, cr and any goose hairpin added to mitfi results

[2021-11-15 16:36:48] Step 9 completed in 0h23m27s

[2021-11-15 16:36:48] Step 10 -- compare_assemblies

[2021-11-15 16:36:49] compare_megahit_msa created

[2021-11-15 16:36:49] Comparing mito_megahit.fasta (17172 bp) and mito_msa.fasta (17151 bp)

Comparison between mito_megahit.fasta (17172 bp) and mito_msa.fasta (17151 bp)

edit distance 21

17149 bp in runs of matches 100 or greater

15976 matches at the beginning and 1173 at end for 17149 contiguous matches

Following describes how to transform the 17172 bp mito_megahit.fasta into the 17151 bp mito_msa.fasta

15976=

1D1=10D1=10D

1173=

Areas of difference between mito_megahit.fasta and mito_msa.fasta

ACTCTAGGCACTTCCATCTAAAACGTATTAATTCTTGTCATCAATTTTTTTTTCAAAAATTTAAAATTTTTTACACATTCCATTCCGCCGATTTCCCTCTTTAATAAAATTCATTCTTTTTTTTTGCAAAAATTTTCTTTTTTTTTGCAAAAATTTTTAC mito_megahit.fasta 15841-16000

||||||||||||||||||||||||||||||||||||||||||||||||||||||||||||||||||||||||||||||||||||||||||||||||||||||||||||||||||||||||||||||||||||||||-|----------|----------| 139 matches 21 indels

ACTCTAGGCACTTCCATCTAAAACGTATTAATTCTTGTCATCAATTTTTTTTTCAAAAATTTAAAATTTTTTACACATTCCATTCCGCCGATTTCCCTCTTTAATAAAATTCATTCTTTTTTTTTGCAAAAATTTT-T----------A----------C mito_msa.fasta 15841-15979

[2021-11-15 16:36:50] Step 10 completed in 0h0m01s

[2021-11-15 16:36:50] Step 11 -- assemble_CR

[2021-11-15 16:36:53] No tandem repeats found in the 99 Control Region sequences and length stddev 0 is less than 20, consensus will be used.

[2021-11-15 16:36:54] Step 11 completed in 0h0m04s

[2021-11-15 16:36:54] Step 12 -- complete

[2021-11-15 16:36:54] msa consensus mitochondrion chosen as best representative

[2021-11-15 16:36:54] mitochondrion.fasta created

[2021-11-15 16:36:55] blastn of features against mitochondrion.fasta for protein coding gene annotation

[2021-11-15 16:36:55] blastn -db hfmt_111521/complete/blastdb/mitochondrion.fasta -query top_match_feature_sequences.fasta -outfmt "6 std staxid stitle qlen qcovhsp qcovus" -task blastn -evalue 1e-10 | sort -k2,2V -k9,9n -k12,12nr

[2021-11-15 16:36:57] mitochondrion.anno created

[2021-11-15 16:36:57] Step 12 completed in 0h0m03s

[2021-11-15 16:36:57] 1h8m13s to complete Mon 15 Nov 2021 04:36:57 PM PST

settings.tsv:

program_title HiFiMiTie version 0.01 -- Find & Analyze Metazoan Mitochondria from HiFi reads

version 0.01

version_date 15-Oct-2021

run_by jdumbacher

mitodb_dir /ccg/blastdbs

mitodb_name mito

taxonomy_dir /ccg/db_sets/taxdump

working_dir *workdir*/pyrocephalus/mito/hfmt_111521

start Mon 15 Nov 2021 03:28:44 PM PST

threads 32

taxid 8782

taxname Aves

taxlineage /ccg/db_sets/taxdump/fullnamelineage.dmp

mitogenomes 964

step 12

code 2

step_completed Mon 15 Nov 2021 04:36:57 PM PST

HiFi_mito_reads 167

top_mito_matches NC_007975

top_mito_match_first_recname Phe_NC_007975

top_mito_match_first_trna F

taxname_trna_starts Aves: F 888, V 32, T 20, I 10, S2 10, E 2, G 1, P 1

taxname_first_trna F

first_trna F

last_trna E

mito_blast_last_trna_counts E:113

trna_order F V L2 I Q M W A N C Y S2 D K G R H S1 L1 T P E

cm_anno_order F 12S V 16S L2 I Q M W A N C Y S2 D K G R H S1 L1 T gh P E

gh_prev_trna T

gh_succ_trna P

gh_rec_count 114

gh_found_in_recs 114

num_CRs 2

Primary_CR CR2

CR1_flanks E F

CR1_mean 178

CR1_stddev 0

CR1_recs_w_CR 113

CR1_type remnant

CR2_flanks T P

CR2_mean 1430

CR2_stddev 0

CR2_recs_w_CR 99

CR2_type Control Region

msa_megahit_edit_dist 21

msa_megahit_edit_cigar 15976=1D1=10D1=10D1173=

CR2_seqs_w_repeats 0

assemble_CR_result msa

anno_order F rrnS V rrnL L2 ND1 I Q M ND2 W A N C Y COX1 S2 D COX2 K ATP8 ATP6 COX3 G ND3 R ND4L ND4

H S1 L1 ND5 CYTB T cr gh P ND6 E cr

finished Mon 15 Nov 2021 04:36:57 PM PST

run_time 1h8m13s

Mitochondrial DNA genome (in fasta text format):

>Pnanus_complete_mitochondrion length 17151

GTCCCTGTAGCTTACAAAAAGCATAACACTGAAGATGTTAAGATGGTTGCCCTAAACACCCAAGGACAAAAGACTTAGTCCTAACCTTACTGTTAGTTCTTGCCACACATATACATGCAA

GTATCCGCACTCCAGTGAAAATGCCCTCGACACCTTAAAAAGATAGTAGGAGCAGGTATCAGGCTCACTTAACTGTAGCCCAAAACGCCTTGCCTAGCCACACCCCCACGGGTACTCAGC

AGTAATTGACATTAAGCAATAAGTGTAAACTTGACTTAGTTAAAGCAACCTACTAGGGTTGGTAAATCTTGTGCCAGCCACCGCGGTCATACAAGAAACCCAAATTAACTTTTCACGGCG

TAAAGAGTGGTCTCTCATTATCACCTCAACTAAGATTGAAATGCAATCAAGCTGTTATAAGCATAGAATGCACTTAACATCCCCTATCAAAATGATCTTAGCCCCCCGACTAATAAAGCC

CACGAAAGCCAGGTCACAAACTGGGATTAGATACCCCATTATGCCTCGCCCTAAATCCTGATGTTTCTCCTACCAAAACATCCGCCCGAGAACTACGAGCACAAACGCTTAAAACTCTAA

GGACTTGGCGGTGCTTCAAACCCACCTAGAGGAGCCTGTTCTATAATCGATAACCCACGATATACCCAACCACTTCTTGCCAAATCAGCCTATATACCGCCGTCGCCAGCTCACCCCTAC

CCTGAGGGCCTAACAGTGAGCATAATAGCCCCCCCGCTAGCAAGACAGGTCAAGGTATAGCTTATGAAGTGGAAGAAATGGGCTACATTTTCTAATATAGAAAACCACCCTACGACAAGG

AACATGAAACTATTCCTAAAAGGCGGATTTAGTAGTAAAGCAGGACAATCATGCCTTCTTTAAACCGGCTCTGGAGCACGTACATACCGCCCGTCACCCTCCTCACAAGCTACTTCACTA

TATTCATACATAATTCACTTTTAAGCTAAAGATGAGGCAAGTCGTAACAAGGTAAGTGTACCGGAAGGTGTACTTAGTCTACCAAGATGTAGCTATAACAAAAGCATTCAGCTTACACCT

GAAAGATACCTGTCCAGTATCAGGTCATCTTGAAGCCTACTCTAGCCCGCTCTTCCATCCACATAAACCAATTGCCCTCTCTTCAAAAACCAAAACATTTTTTTACCTCAGTATAGGCGA

TAGAAAGGTACCCCAAGGAGCAATAGAGACCACGTACCGCAAGGGAAAGATGAAATAGTAATGAAAACCCAAGCGCCAAATAGCAAAGTTCAATCCTTGTACCTTTTGCATCATGATTTA

GCAAGAACTGACCAAGCAAAACGCTAATTTAAGCTTGCCACCCCGAAACCCGCGCGAGCTACTTACGAGCAGCTATTCAATGAGCAAACCCGTCTCTGTTGCAAAAGAGTGGGACGACTC

GTCAGTAGAGGTGAAAAGCCAATCGAGCCGGGCGATAGCTGGTTGCCTACGAAACGAATCTTAGTTCACCCTCAATCTCCCTCCAAGGCCCAAACCAAACCCCCATGAAGAAGATCGAGG

GCTACTTAAAGGAGGTACAGCTCCTTTAAAAAGAACACACTCTCCACAAGCGGATAAGTTCTCCCATATCCTAACTGTGGGCCTTAGAGCAGCCACCACCAAAGAATGCGTCAAAGCTCA

ACCCCTAAAAATTTGAAAGCCCTACAACTCCCTCTCCCCTAATAGGCTAACTTATATCTATAAGAGAATCAATGCTAAAATGAGTAACTAGGAACCTCCTCTATGGCGTAAACTTACATC

CCCACATTATTACCAAATTTAGAGGTATATACTACCCTAACAAGACCCAATACCATAAAACCTGTTAACCCACCCAAGGAACGCCCCAAAGAAAGATTAAAATCTGCAAAAGGAACTAGG

CAACTCCAAGGCCCGACTGTTTACCAAAAACATAGCCTTCAGCGAGTCAAGTATTGAAGGTGAAGCCTGCCCAGTGACAGTACGTTCAACGGCCGCGGTATCCTAACCGTGCGAAGGTAG

CGCAATCAATTGTCCCATAAATCGAGACACGTATGAATGGCTAAACGAGGTCTTAACTGTCTCTTGCAGATAATCAGTGAAATTGATCTCCCTGTGCAAAAGCAGGGATCATAACATAAG

ACAAGAAGACCCTGTGGAACTTCAAAATCAACAGCCACTCCCATATACCACTCCCCCTACATATGGAATACCTCACGGGACTCTATTGGCCTGTATTTTTTGGTTGGGGCGACCTTGGAG

AAAAACGAATCCTCCAAAAATTAGACCACCCCTCTACACCAAGAACAACCTATCAACGTACTAACAGTAACCAGACCCAATACCATTGATTAATGGACCTAGCTACCCCAGGGATAACAG

CGCAATCTCCCCCAAGAGCCCCAATCGACAGGGAGGTTTACGACCTCGATGTTGGATCAGGACATCCTAGTGGTGCAGCCGCTACTAAGGGTTCGTTTGTTCAACGATTAACAGTCCTAC

GTGATCTGAGTTCAGACCGGAGTAATCCAGGTCGGTTTCTATCTATGATAAACTCCTTCCAGTACGAAAGGACAGAAAGAGTAAGGCCAATACCCCAAGCACGCCTTCGCCCAAAGATAT

GAACTCAACTAAATCTCTTAGGGCTAACTTTCCTTACCCTCCTAAAAAAGGACCGCTAGAGTGGCAGAACTTGGCAAATGCAAAAGGCTTAAGCCCTTTATCTAGAGGTTCAAATCCCCT

CTCTAGCTTCCACACCAACTATATGATAATTTACCCCAACCTAACTCATCTCATCATATCCTTATCCTATGCCTTACCCATTCTAGTTGCCGTAGCCTTCTTAACCCTAGTAGAACGTAA

AGTCCTAAGCTATATACAAACACGTAAAGGCCCAAACATCGTAGGACCATTTGGTCTTCTACAACCTATAGCAGATGGTGTAAAACTTTTTATTAAAGAGCCAATCCGCCCATCCACTTC

TTCCCCCTACCTATTTATTCTCACTCCAATATTGGCCCTCCTTCTAGCAGTCATAATCTGAACCCCACTCCCACTACCTTTTCCTCTTGCAGATATAAACCTAGGCATACTTTTCCTCCT

CGCACTATCTAGCCTCGCAGTCTATTCAATTCTATGATCTGGATGGGCCTCAAACTCAAAATATGCACTAATCGGTGCTCTACGAGCAGTCGCACAAACAATTTCCTACGAAGTCACATT

AGCCATTATTCTCCTATGTATTATCATTTTTAGCGGAAACTATACCCTTAATACCCTTACTACAACCCAAGAACCAATATACCTCATATTCTCTTCTTGACCCCTAGCAATAATATGATA

TATTTCCACCCTCGCAGAAACAAACCGTGCCCCATTTGATCTAACAGAGGGTGAATCAGAACTAGTCTCAGGGTTTAACGTTGAATATGCTGCAGGACCCTTTGCCTTATTTTTCCTAGC

TGAATACGCTAACATCATACTTATAAATGCACTAACTACTATCTTATTCTTAAACCCAAGTTCACTTAACATCACCCAAGAACTCTACCCACTAACTCTAGCTACTAAAACCCTCCTACT

TTCCGCAGGATTCCTATGAATCCGCGCCTCCTACCCTCGATTCCGCTACGACCAACTCATGCACCTACTATGAAAAAGTTTCCTGCCACTTACACTATCATTATGCCTATGACATATCAG

CCTTCCAATCTCATTCGCAGGTCTACCACCTCACCTAAGACTCCCGAGGAAATGTGCCTGAACGTTAAAGGGTCACTATGATAAGGTGAACATAGAGGTATACCAACCCTCTCATTTCCT

AGACTTAGAAAAGTAGGAATCGAACCTACACAGAAGGAATCAAAATCCTTCATACTTCCTTTATATTATTTCCTAGTAAGGTCAGCTAATCAAGCTATCGGGCCCATACCCCGAAAATGA

TGGTTTAACTCCTTCCCTTACTAATAAACCCCCAAGCTAAATTCATTTTCTCAATAAGTCTCCTCCTAGGTACTACAATTACAATTTCAAGCAACCACTGAATTATAGCATGAGCTGGAC

TTGAAATTAATACTTTAGCTATCCTCCCCCTAATCTCAAAATCTCATCACCCACGAGCCATTGAAGCCTCAACTAAATACTTCCTAGTCCAAGCAACCGCCTCCACACTCCTCCTATTTT

CCAGTATAACCAACGCATGATTCACTGGTCAGTGAGATATTACTCAACTCACTCATTCAGTATCATGTCTACTGCTAACAGCTGCAATCTCAATAAAGCTAGGCTTGGTCCCATTCCACT

TCTGATTCCCAGAAGTCCTTCAAGGTTCCTCCTTAATAACTAGCCTGCTGCTAGCCACAATCATAAAATTCCCTCCCACCGTACTCCTCTTGTTAACCTCCCCCTCACTAAATCCCACAC

TACTATCCATATTAGCAATTGCCTCCGCTGCCCTAGGAGGCTGAATAGGGCTCAACCAAACTCAAATCCGCAAAATTATAGCCTTCTCGTCTATCTCCCATCTAGGCTGAATGACCATTA

TCCTCATCTACAACCCTAAACTCACGCTTATTGCTTTCTACCTCTATTCTCTAACTACAGCTGCCATTTTCTCTGCCCTCAGTGCTATCAACTCTTTAAAACTGACCACCTTAATAACTG

CGTGATCCAAAATTCCTGCACTAAGTGCAACCCTAATACTAACTCTTCTATCTCTCGCAGGTCTTCCCCCATTAACTGGATTTCTACCAAAATGGCTAATTATTCAAGAGCTAACTAAAC

AAGAACTAACAGCCACAGCTACTATCATTGCCCTACTCTCTCTCCTAGGACTTTTCTTCTACCTCCGTCTTGCTTACTGTGCAACAATTACACTTCCCCCAAACTCTGCTAACCACATAA

AACAATGACGAACCAACAAGCTCACTAACTTACTAACCCCCACCCTCATTATAATATCAGCTACACTTTTACCCCTATCACCTACAATCCTTACTATTCCATAGAAGCTTAGGTTACTTA

AACCGAAGGCCTTCAAAGCCTTAGACAAGAGCTATTTCCTCTTAGCTTCTGCTAAGATCCGCAGGATACTAACCTGCATCCTCTGAATGCAACCCAGATGCTTTAACTAAGCTAGGACCT

TCCCTAGACAGATGGGCTTCGATCCCATAACACTCTAATTAACAGCTAGATGCCATAACCTACAGGCTCCTGTCTACTAGACTCCGGTATATACTTAATACACATCTTTGAGTTTGCAAC

TCAATATGAAATTCACTACAGAGTCGATAAGAAGAGGAATTAAACCTCTGTCAAAAGGACTACAGCCTAACGCTTCAACACTCAGCCATCTTACCTATGACCTTCATTAACCGATGATTA

TTCTCAACCAACCATAAAGACATTGGCACATTATACCTAATTTTTGGCGCTTGAGCTGGCATAATTGGTACCGCCCTAAGCCTCCTTATCCGAGCAGAATTAGGACAACCAGGGACTCTC

CTAGGGGATGACCAAATCTATAATGTAATCGTCACTGCTCATGCCTTTGTAATAATCTTCTTTATAGTAATGCCCATTATAATCGGAGGATTCGGTAATTGATTAGTTCCCTTAATAATT

GGTGCCCCCGACATAGCATTCCCCCGCATGAACAACATAAGTTTCTGACTATTGCCCCCATCATTCCTCCTTCTTCTAGCCTCATCCACAGTCGAAGCCGGAGCCGGAACAGGATGAACT

GTCTACCCACCATTAGCTGGAAACCTAGCACATGCTGGAGCTTCCGTAGACTTAGCTATTTTCTCCCTTCACCTTGCAGGTGTCTCTTCAATTTTAGGTGCCATCAATTTTATTACTACC

GCAATTAACATAAAACCACCCGCCCTATCACAATACCAAACTCCCCTCTTTGTGTGATCCGTCCTAATCACTGCAGTCCTTCTCCTCCTCTCTTTACCAGTCCTTGCCGCAGGTATCACC

ATGCTATTAACAGATCGCAACCTTAACACTACATTTTTCGACCCCGCAGGAGGAGGAGACCCAATCTTATACCAACATCTTTTCTGATTCTTTGGTCACCCCGAAGTTTATATTCTAATC

TTACCAGGATTCGGTATTATTTCTCATGTAGTAGCATACTACGCTGGCAAAAAAGAACCATTCGGCTACATAGGAATAGTCTGAGCCATACTCTCTATTGGTTTCCTAGGTTTTATCGTA

TGAGCTCATCATATATTTACAGTAGGAATGGACGTAGACACTCGAGCATACTTTACATCCGCCACAATAATCATTGCAATCCCTACCGGCATTAAAGTTTTTAGCTGACTAGCTACACTT

CATGGAGGGACTATTAAATGAGACCCTCCCATGCTATGAGCTCTGGGATTTATCTTTCTATTTACCATCGGAGGACTCACAGGCATTGTCTTAGCCAACTCTTCCTTAGACATTGCTCTT

CATGACACTTATTATGTAGTAGCCCACTTCCACTATGTCTTATCAATAGGGGCTGTATTTGCAATCCTAGCAGGCTTTACACACTGATTCCCACTCTTCACAGGCTACACCCTCCATCAA

ACATGAGCTAAAGCCCATTTTGGAGTCATGTTTACAGGTGTAAATTTAACCTTCTTCCCCCAACACTTCCTAGGCCTAGCTGGTATACCACGACGATATTCAGATTACCCAGATGCCTAC

ACCTTATGAAACACCCTATCCTCTATTGGCTCTCTCATCTCTATAACTGCTGTAATTATGCTTATGTTTATCATCTGAGAAGCTTTTGCATCCAAACGAAAAGTAATGCAACCTGAACTT

ACTTCTACCAACATTGAATGAATCCATGGTTGCCCTCCCCCATATCACACTTTTGAAGAACCAGCCTTCGTCCAAATCCAAGAAAGGAAGGAATCGAACCCTCACAAGCTGGTTTCAAGC

CAACCGCATTAAGCCACTTATGCTTCTTTCTTATGGGTTGTTAGTAAAAAAATTACATAGTCTTGTCAAGACTAAATCACAGGTGAAAACCCAGTACACCCCATCCCTTCCAATGGCCAA

CCACTCCCAATTCGGTTTCCAAGACGCCTCATCTCCCATCATAGAAGAATTAATTGAATTCCATGATCACGCTTTAATAGTTGCCCTAGCAATCTGTAGCCTAGTACTTTACCTCCTGAC

CCTTATACTCATAGAAAAACTATCATCCAACACTGTTGACGCGCAAGAAGTAGAACTAGTCTGAACCATTCTACCCGCAATTGTCCTAATCATGCTAGCCCTTCCCTCACTTCAAATTCT

TTATATAATAGACGAAATTGACGAACCCGACCTCACACTTAAAGCTATCGGCCACCAATGATATTGATCCTATGAATATACAGACTTCAAAGAATTATCCTTCGATTCATACATACTACC

AACTACAGAACTCCCACTAGGTCACTTTCGACTACTAGAAGTTGACCACCGTATTGTTATTCCTATAGAATCCCCCATTCGGGTTATCGTAACAGCCGATGACGTCCTACATTCTTGAGC

CATTCCTAGTCTCGGCGTAAAAACTGACGCAATCCCAGGGCGACTCAACCAAACATCATTTATTACTACCCGACCTGGAATTTTCTATGGTCAATGTTCAGAAATCTGCGGAGCGAACCA

TAGCTTCATACCTATCGTAGTTGAATCAGCCCCCCTAACCCACTTCGAAAGCTGATCTTCACTCCTATCATCATAAACATTAAGAAGCTATGAACCAGCACTAGCCTTTTAAGCTAGAGA

AAGAGGACCTCAACCCTCCTTAATGGTATGCCACAGCTAAACCCAGCACCATGATTCTTTATCATGTTATCCTCATGATTTATTTTTTCCTTTATCCTTCAGCCCAAAATTCTATCATTC

ACCTCCACAAACCAACCCTCTAACAAAGCCCTCTCTACCATAAAAACATCCCCCTGAAACTGACCATGAACCTAAGCTTCTTTGACCAATTTATAAGCCCTTCCCTATTAGGAATCCCAT

TAATCCTTATCTCACTCTTGTTTCCAACACTCCTATACCCTTCCCCTAGCAACCGATGAATTACAAACCGTATTTCTACCCTCCAACTCTGACTCTTTCAACTACTCACAAAACAACTTA

TAACCCCAATAAACAAAAAAGGACATAAATGAGCCCTAATCTTACTCTCACTCATAATCTTTCTCCTGACAATTAATCTTCTAGGGCTCCTACCTTATACATTCACTCCTACTACCCAAT

TATCCATAAACCTAGCCCTAGCCTTTCCACTATGACTCGCAACCTTACTCACAGGCCTACGAAACCAACCCTCAGCCACTCTAGGCCATCTCCTGCCAGAAGGCACCCCCACACCCTTAA

TTCCTGCCCTCATTTTAATTGAAACTACTAGCTTACTTATTCGCCCCTTAGCCCTAGGAGTTCGCCTTACTGCTAACCTCACAGCCGGACATTTACTCATCCAACTTATTTCAACTGCTA

CTACTGTCCTTCTACCTATAATTCCAACAGTATCTTTACTTACTGCAACCATCCTCTTTTTACTCACTATCTTAGAGGTAGCTGTTGCCATAATTCAAGCCTACGTTTTCGTTCTACTAT

TAAGTCTTTACCTACAAGAAAATATCTAAACCCTAATGACCCACCAAGCACACTCATTTCATATAGTAGATCCAAGCCCCTGACCCATTTTTGGAGCAGCAGCTGCTCTTCTCACTACTT

CAGGACTAACCATATGATTTCATCATAACTCTACCCAACTCCTAACCTTAGGGTTATTATCCATAGTCCTTGTTATAATTCAATGATGGCGCGATATCGTACGAGAAAGCACATTCCAAG

GCCATCATACTCCTACCGTCCAAAAAGGTCTACGTTACGGTATAATCTTATTTATTACATCCGAAGCATTTTTTTTCTTAGGCTTCTTCTGGGCATTCTTCCATTCAAGCCTAGTCCCAA

CTCCAGAACTCGGAGGACAATGACCTCCTATAGGAATCAAACCCCTCAACCCCATAGAAGTTCCCCTATTAAATACAGCGATCCTACTAGCCTCTGGTATTACCGTCACATGAGCACACC

ATAGCATTGTAGAAGCCAACCGAACCCAAGCAACCCAAGCTCTATTTATAACTATCGCGTTAGGATTCTACTTCACAGCTCTCCAAGCAATAGAATATTATGAAGCCCCATTTTCAATCG

CCGATGGAGTCTACGGATCAACTTTTTTTGTAGCCACAGGATTTCATGGCCTTCATGTAATCATTGGTTCTTCCTTCCTATCCGTCTGCTTCCTTCGACTTGTTAAATTCCACTTCACCT

CTAACCACCACTTTGGTTTCGAAGCAGCAGCTTGATACTGACATTTTGTTGATGTCATCTGACTATTCCTCTATATAACTATTTACTGATGAGGATCCTGCTCTTCTAGTATAATAATTA

CAATTGACTTCCAATCTTTAAAATCTGGACCAAAACCAGAGAAGAGCAATTAACATAATCACCTTCATACTGATATTATCCCTATCACTAAGTATCATCCTCACTACACTAAATCTATGA

CTAGCACAAACCAACCCAGACTCAGAAAAATTATCCCCCTACGAATGCGGCTTCGACCCACTAGGATCCGCTCGACTCCCATTTTCAATCCGATTCTTTCTAGTCGCAATTTTATTCCTC

CTCTTTGACTTAGAAATTGCACTCCTTTTACCCCTCCCGTGAGCTACTCAACTTCAATCCCCCCTCACCACCCTAACATGGGCATTTATCATACTTCTTCTCCTAACACTAGGACTTATA

TATGAATGAGCCCAAGGAGGTCTAGAATGGGCAGAATAACTCCAGAAAGTTAGTCTAATAAAGACAGTTGATTTCGGCTCAACAAATCACAGTCCTACCCTGTGACCTTCTCTATGACCT

TCCTACACCTAACTTTCTATTCCACTTTTACCCTAAGCAGCCTAGGCCTAGCCTTCCACCGAACCCACCTAATCTCTGCCCTTTTATGCTTAGAAAGCATAATACTGTCTATATATATCG

CCCTTTCTTTATGACCCATCCAAACACAAATATCCTCCTCCACCCTAATACCCATCCTAATATTAGCATTCTCTGCCTGCGAAGCAGGCGCTGGCCTTGCAATTCTAGTAGCCTCAACCC

GGACCCATGGATCCGACCACCTCCACAACCTCAACCTCTTACAATGCTAAAAATTATCATCCCAACGATAATACTCCTCCCAACAGCCCTCCTATCACCCTCAAAGTCCCTATGAATCAA

TGTTACCTCCCACAGCCTATTAATTGCAGCCGTAAGCCTCCATTGACTCTCCCCAACATACTACGCTAACAAAAATCTATCTCAATGACTTGGGGTGGATCAAATCTCATCACCATTACT

AGTCTTATCTTGCTGACTTCTACCCCTCATAATATTAGCTAGCCAAAACCACCTTCAACAAGAACCATCCACACGAAAACATATTTTTATCCTAACCATAATTCTTACTCAACCCTTTAT

TTTACTAGCATTCTCATCCCTAGAACTCATATTATTTTATATTGCATTCGAAGCTACCTTAATCCCCACCCTCATCCTAGTTACACGGTGAGGAAGTCAACCAGAACGCCTAAGTGCTGG

TATTTACCTACTATTTTACACACTTATTAGCTCCCTCCCACTATTAGTTGTTATCCTCCACCTTCATACCCAAACGGGCACCATGCATCTTGCTATAATAAAACTTATCCACCCGTCCCT

TACTGACTCATGAACAGGAACTTTATCCAGTCTCGCCTTACTCCTTGCATTCATAGTAAAAGCTCCTCTTTACGGACTACACTTATGACTTCCAAAAGCACACGTAGAAGCCCCAATCGC

AGGATCTATGCTTTTAGCCGCCCTCCTTCTAAAACTTGGAGGATATGGCATCATACGCGTATCCATAATTACAAACCCCCTCCTAAACTACCTACACTACCCATTTCTATCTCTAGCTCT

ATGAGGAGCATTAATAACAAGCTCCATCTGTCTCCGCCAAATCGACTTAAAATCTATAATCGCATATTCTTCTGTAAGCCACATAGGCCTAGTCGTAGCCGCAGTCATAATCCAAACCCA

CTGATCATTTTCAGGGGCTATAATCCTAATAATCTCCCATGGACTTACATCCTCCATATTATTCTGTCTAGCCAACACAAACTATGAACGAACACATAGCCGAATTCTATTCCTAACACG

AGGCCTACAACCCCTCCTTCCCCTCATAGCTATCTGATGACTTCTAGCCAGTCTCACTAACATAGCACTTCCCCCCACAACCAACCTTATAGCCGAACTAACCATTATAATTGCCCTCTT

TAACTGATCTGCTCTCACCATTATCTTAACCGGAATTGCAACCCTCTTAACTGCCCTATACACCCTATACATGTTACTTACAACCCAGCGAGGCCCCATCCCAACTTACATGACATCAAT

TCAAAACTCCAACACCCGAGAACACTTACTTATAACTCTCCACATCCTCCCCGCCCTTTTACTTATTCTAAAACCAGAACTTATCTCAATAATCCCTTCATGCAAGCATAGTTTTAATCC

AAACATTAGACTGTGATCCTAAAAATAGAAGTTAAACTCTTCTTGCCTGCCGAGGGGAGGTTTAACCAACAAGAACTGCTAACTCTTGCATCTGAGTATAAAAGCTCAGCCCCCTTACTT

TTAAAGGATAGTAGCAATCCACTGGTCTTAGGAGCCATTTACCTTGGTGCAAATCCAAGTAAAAGTAGTGCATATCCCATTAATCCTAAACTCCTTCATAATCCTCACCCTTCTCATCCT

ACTAACCCCAATCTTCCTTCCCCTTCTAATCAAAAACTACCAAAACTCTCCAACTAACATTACACAAACTGTCAAAGCCTCATTTATTACCAGCCTAGTGCCAATAACTCTTTTCATATA

TATAAACATGGAAAGTATTTCATTATACTGAGAATGAAAATTTATTATAAACTTCAAAATCCCACTAAGCTTCAAAATAGACCAATACTCACTAACATTCTTTCCCATCGCATTATTTGT

AACATGATCTATTCTTCAATTTGCATCATGATACATAGCCTCAGAACCCTTCATCACAAAATTCTTCTTCTATCTTCTAACCTTTCTTATCGCTATACTTTGCCTCATCATCGCAAACAA

CATATTCCTTTTATTTATTGGATGAGAAGGAGTCGGAATCATATCTTTTCTCCTTATCGGATGATGACACGGACGAGCAGAGGCCAACACAGCTGCTCTCCAAGCCGTACTCTATAACCG

CATCGGAGACATTGGCCTAATCCTATCAATGGCCTGACTAGCTTCCTCCCTAAACACCTGAGAAATTCAACAACTGTCATTTCAGAATCAAATTCCACTACTCCCCCTTCTAGGCCTAAT

CCTCGCAGCCACAGGAAAATCCGCTCAATTCGGTCTACATCCATGACTCCCAGCAGCCATAGAAGGCCCCACTCCTGTCTCCGCCCTACTTCATTCTAGCACTATAGTAGTAGCTGGAAT

CTTCTTACTTATCCGCACCCACCCCATACTTTGCAACAACCAAATTGCCCTTACTTTGTGTCTCTGCCTGGGAGCTCTCTCCACACTATTTGCCGCCGCATGCGCTCTTACCCAAAATGA

CATCAAAAAAATCATCGCCTTCTCCACATCAAGCCAATTAGGCCTAATGATAGTAACAATTGGCCTAAACCTTCCACAATTAGCCTTCCTACACATTTCAACTCATGCATTCTTCAAAGC

AATACTCTTCTTATGCTCAGGCTCAATCATTCATAGCCTCAACGGTGAACAAGACATTCGAAAAATAGGAGGCCTACAAAAATCACTTCCAATCACCATATCATGCTTAACCATTGGCAA

CCTAGCCCTCATAGGAACCCCCTTCTTAGCAGGATTCTACTCAAAAGACCTTATCATCGAAAACCTAAACACATCTTACCTCAACACGTGAGCACTTCTCTTAACACTCTTAGCCACATC

CTTTACTGCAACTTACAGCATACGCATATCCTTACTAGTACAAACCAACTTCACCCGAACTTCCTCCTCCACTCCAATCAATGAAAACCACCCAGCAGTCATAAACCCAATTAGCCGCCT

TGCCATTGGAAGCATCATAGCCGGACTACTAATCACCTCCTTTATCCTTCCTACAAAAACTCCACCTATAACTATATCTTTTACTACAAAAACTGCAGCCATCATTGTATCCCTACTAGG

AATCATAATTGCACTAGAACTATCAAAACTAACCCATATTCTCACTAGCCCTAAACAAAATGCCACTACTAACTTCTCTACCTCCTTAGGCTACTTCAACCCCCTAATACACCGCCTAAG

TTCAACCAAACTACTAAGCACCGGACAAAATATCGCATCACACCTAATCGACCTGTACTGGTATAAAAAAATAGGCCCCGAAGGACTCGCAACCCTCCAACTTAAAGCTACCAAAACCTC

AACTACTCTCCACAGCGGATTAATCAAAACTTACCTAGGATCCTTCGCACTATCTATTTTTATCATTATCCTGTACATACACGGAACTAATCAATAATGGCACCCAACCTACGAAAACAC

CACCCTCTCCTAAAGATAGTAAACGACTCTCTTATCGATCTTCCCACTCCATCAAACATCTCAGCCTGATGAAACTTTGGTTCTCTTCTAGGAATCTGCCTAGCAACACAAATCGTCACC

GGCCTCCTACTTGCTATACACTACACTGCAGACACCTCTTTAGCCTTTACATCAGTTGCCCACACATGCCGAAACGTCCAATTTGGCTGACTAATCCGTAACCTCCATGCAAATGGAGCA

TCCTTTTTCTTCATCTGCATTTACCTACATATCGGGCGAGGATTCTACTATGGATCATACTTATATAAAGAAACCTGAAACACTGGCGTTATCTTACTTCTAACCCTAATAGCAACTGCT

TTCGTCGGATATGTCCTTCCATGAGGGCAAATATCATTCTGAGGCGCTACAGTAATTACCAATTTATTTTCAGCAATCCCTTACATCGGTCAAACACTCGTAGAATGAGCCTGAGGTGGA

TTTTCAGTTGATAACCCTACACTTACTCGATTCTTTGCCCTCCACTTCCTTCTCCCATTCATAATCGCAGGTCTTACATTTATCCACCTAACCTTCCTTCATGAAACAGGATCAAACAAC

CCTTTAGGAATTTCCTCAAACTGCGATAAAATTCCATTCCACCCTTATTTTTCCACAAAAGATATCTTAGGCTTCATTATTCTACTCTTACCACTAATAACATTAGCTATATTCTCACCT

AATCTCCTAGGCGACCCAGAAAATTTTACGCCCGCCAACCCATTAGTAACTCCCCCCCACATTAAACCAGAATGATATTTCCTTTTTGCGTATGCTATCCTTCGATCCATCCCTAACAAG

CTCGGAGGGGTCTTAGCACTTGCAGCCTCAGTCCTAGTACTATTTCTAGTCCCATTCCTTCACATATCGAAACAACGCACCATAACCTTCCGCCCTCTCTCCCAACTACTATTCTGAATC

CTAGTAACTAACCTCCTCATCCTAACATGAATTGGAAGCCAACCAGTAGAACATCCATTCATTATTATCGGCCAACTGGCCTCACTAACTTACTTCACTATCATTCTAATCCTATTCCCA

ATTATCGAAATACTAGAAAACAAGCTACTAAGTTTCTAACTACTCTAATAGTTTATTAAAACATTGGTCTTGTAAACCAAAGACTGAAGGTTCCCCCCTTCTTAGAGTTATAAACCCCTA

CAACTTAACACCAGCACACACTTCCCTCTAATAATAAAAGGGACCCCCCCCCTCCCCCCCAATTTTTACATTTTAGGGTATGTATTACTTTGCATTACATTATTTTCCACATTAGACATA

CCATGCATGTAGGAAAATGTCATATTAATTTAATGGTCGGAGCGCATAAATTTTCATGCTTAGTCCCATAACAATCCACCCAAGCCATATCCCGATCTAGGCACATTTCTACTTCAAGGA

CCCGCCATGTCATGATCTAGGAATATTCCCAATACCCGGACTAAAACCTATTAAATGCCAGTTTTTGCATAAATCCTTCTTATACACGAGGAACCTCCCAAGACTTAAAATCCATGTACT

AAGGACCACTCATCAATTAACCTTGCTCTACGTACTGACCAAGAAGTACTGGGTTATTTATTGATCGTACCCCTCACGAGAAACCAGCAACCCGGCGTTAGTAATGTTTATCACGACCAG

CTTCAGGTGCATTCTTCCCTCGTCCCTGGCCCAACTTGCGCTTTTGCGCCTCTGGTTCCTCGGTCAGGGCCATAACTTGATTAATTTTCCTATACTTGCTCTCCGTTACTAATGGTTGGG

GATGCTTGACCATTATGGACCTCGTGATCGCGGCATCTTCTTTCTTCTATACTTCTCTTATCTTTTTTGGGGTAGATCTCAATAAGCCCTTCAGAGTGCTCCGCCAGTGGGCCCCCATAT

ATGCTTGACATGTCCATCTTGTGGTCGGCGTGCGGTTTTCTCACTTTCTCGAGCAAATTAATGATATGGGGTCATGTGTAACTCGGTCTCATACTGTAGCACTGATGCACTTTGTTGTGC

TATTGGTTTGGAACTTCCAGTTCGTCTCTAAGCTAGGTGTTGTTCAGTTAATGCTCGCCGGACATATTTTGACCTCGTCAATTTCACAAAAATTGACAAAAATTTATACATTTTGCCACT

ACTCTAGGCACTTCCATCTAAAACGTATTAATTCTTGTCATCAATTTTTTTTTCAAAAATTTAAAATTTTTTACACATTCCATTCCGCCGATTTCCCTCTTTAATAAAATTCATTCTTTT

TTTTTGCAAAAATTTTTACATTCAATTTAAAAACCCATGAATTTTCTAGGCTAATCCCTTTTATTATCATCGTTTTATGTTTATTTATTTATTTTTGCATAACCATCCATCAATTTTTCT

ACTAGAATCCCTTTAATCAACGAATTATTCAACTTACCCCCACAAAATTTCCAACGATATATTTATTGTTTTGACCCTTTCACTACCCTAACAATCAACCCCTTAACCGATTTTATTCAA

ACCTAACTATGAGACTAACACAACCCAATCATCTTACTCCATCCCATATAATCATCAATTAAACTACCAGTTTAAACTTGTTCAACCCCCCCCCCACATTCAGAGAAAAAGGAATCAAAC

CTTTATCATCAACTCCCAAAGCTGACATTTTAATTAAACTATTCTCTGATTTATCTCCCCTAAACAGCCCGAATAGTCCCCCGAGACAGCCCTCGCACAAGCTCCAAAACCACAAATAAA

GTTAATAACAACCCCCAACCCGCAACAAAAAACTGTCCCACTCCCCAAAAATAAAACATAGCTGCACCATCAAAATCTAACCGTACAGAAGCCATCCCCTCACAATCCACTGTACCGACA

CCAACCAACCACCCCCCCGGCAACCCAACTAAAAACACCCCACATACTAATACTACAACTAACCCTACAGCATATACTAAAACTCGTCAATCCCCCCAAGCCTCAGGAAAAGGATCAGCC

GCCAATGATACTGAGTAAACAAACACTACCAACATTCCGCCTAAATATACTAAAAATAGTACCAACGATACAAAAGACGATCCTAAACTCATCAACCACCCACACCCCACAATAGACCCC

AACACCAGACCTATCACCCCATAATAAGGAGAAGGATTAGAAGCTACCGCCAATCCTCCTAAAATAAAACAAATACTTAAAAAAATCATAAAGTATGTCATAGAATTCCTACTTGGTTTC

TCTCCAAGACCTATGGCCTGAAAAACCACCGTTGTAAATTCAACTATAAGAACTTACATTACTACAAACTCCTAACCAAACACAACCAAAACTTCTTCCACATCACCTTACTTACTACTT

TCAACCTCCCCAACCCATTCTAACAAATGCTTCTTTTTTTTAGACCCATCCAATACAACCAATACCCCTACGTCAAAAGTACTACAACTATAAAACCAACACACAACTAAA

**Part II. Nuclear genome assembly and annotation**

**Estimates of genome completeness using BUSCO and compleasm, passeriformes lineage.**

Completeness of genome assembly based upon BUSCO v5.4.7 analyses:

C:96.2%[S:95.9%,D:0.3%],F:0.6%,M:3.2%,n:10844

10437 Complete BUSCOs (C)

10400 Complete and single-copy BUSCOs (S)

37 Complete and duplicated BUSCOs (D)

62 Fragmented BUSCOs (F)

345 Missing BUSCOs (M)

10844 Total BUSCO groups searched

Completeness of genome assembly based upon compleasm v0.2.2 results:

C:99.45%[S:99.35%, D:0.09%],F:0.30%,M:0.25%,n:10844

10784 Complete BUSCOs (C)

10774 Complete and single-copy BUSCOs (S)

10 Complete and duplicated BUSCOs (D)

33 Fragmented BUSCOs (F)

27 Missing BUSCOs (M)

10844 Total BUSCO groups searched

Completeness of genome assembly based upon combined BUSCO and compleasm analyses:

C:99.72%[S:99.63%,D:0.09%],F:0.-6%,M:0.22%,n:10844

10814 Complete BUSCOs (C)

10804 Complete and single-copy BUSCOs (S)

10 Complete and duplicated BUSCOs (D)

6 Fragmented BUSCOs (F)

24 Missing BUSCOs (M)

10844 Total BUSCO groups searched

BUSCO reported 345 missing, while compleasm found all but 27, of the 10844 passeriformes lineage single copy orthologs. Of the 27 not found by compleasm, 3 of those however were found by BUSCO.

Table S1. *P.nanus* 1.0 Repeat Elements

| file name: bPyrNan1.0.fasta | | | |
| --- | --- | --- | --- |
| sequences: | 152 | | |
| total length: | 1072479546 bp (1072468046 bp excl N/X-runs) | | |
| GC level: | 42.30% | | |
| bases masked: | 120094435 bp (11.20 %) | | |
|  | number of elements* | length occupied of sequence | percentage |
| Retroelements | 223983 | 80090817 bp | 7.47% |
| SINEs: | 9529 | 1115561 bp | 0.10% |
| Penelope: | 168 | 60923 bp | 0.01% |
| LINEs: | 180191 | 58469783 bp | 5.45% |
| CRE/SLACS | 0 | 0 bp | 0.00% |
| L2/CR1/Rex | 172099 | 56473377 bp | 5.27% |
| R1/LOA/Jockey | 0 | 0 bp | 0.00% |
| R2/R4/NeSL | 4414 | 330241 bp | 0.03% |
| RTE/Bov-B | 1812 | 1249014 bp | 0.12% |
| L1/CIN4 | 78 | 18471 bp | 0.00% |
| LTR elements: | 34263 | 20505473 bp | 1.91% |
| BEL/Pao | 0 | 0 bp | 0.00% |
| Ty1/Copia | 315 | 128833 bp | 0.01% |
| Gypsy/DIRS1 | 2077 | 1374185 bp | 0.13% |
| Retroviral | 31601 | 18935339 bp | 1.77% |
|  |  |  |  |
| DNA transposons | 26638 | 5513805 bp | 0.51% |
| hobo-Activator | 4104 | 921735 bp | 0.09% |
| Tc1-IS630-Pogo | 570 | 98656 bp | 0.01% |
| En-Spm | 0 | 0 bp | 0.00% |
| MULE-MuDR | 359 | 165393 bp | 0.02% |
| PiggyBac | 0 | 0 bp | 0.00% |
| Tourist/Harbinger | 8018 | 827140 bp | 0.08% |
| Other (Mirage, P-element, Transib) | 0 | 0 bp | 0.00% |
|  |  |  |  |
| Rolling-circles | 217 | 74483 bp | 0.01% |
|  |  |  |  |
| Unclassified: | 43282 | 16674470 bp | 1.55% |
|  |  |  |  |
| Total interspersed repeats: |  | 102340015 bp | 9.54% |
|  |  |  |  |
| Small RNA: | 2576 | 410422 bp | 0.04% |
|  |  |  |  |
| Satellites: | 3630 | 1299929 bp | 0.12% |
| Simple repeats: | 278611 | 13149163 bp | 1.23% |
| Low complexity: | 54796 | 3038055 bp | 0.28% |
|  |  |  |  |

Table S2. Scaffold composition: assignment to chromosome and information about size and gene and telomere content.

| Scaffold /Chr number | Size bp | Location along genome | Cumulative Genome percentage | BUSCO markers data into categories of total BUSCOs (B), Complete and single-copy (C), Complete and duplicated (D), Fragmented (F), and any duplicated, fragmented or complete (d). | Telomere regions present in the scaffold |
| --- | --- | --- | --- | --- | --- |
| Chr1_Lvf | 117695848 | 117,695,848 | 10.97% | B:890 C:886 F:1 D:2 d:3 | telomeres: BOTTOM |
| Chr2_Lvf | 115308024 | 233,003,872 | 21.73% | B:904 C:903 F:1 D:0 d:0 | telomeres: TOP BOTTOM_near BOTTOM_near |
| Chr3_Lvf | 97861697 | 330,865,569 | 30.85% | B:577 C:575 F:0 D:1 d:2 |  |
| Chr4_Lvf | 74117589 | 404,983,158 | 37.76% | B:659 C:658 F:1 D:0 d:0 | telomeres: BOTTOM |
| ChrZ_Lvf | 74110192 | 479,093,350 | 44.67% | B:536 C:534 F:0 D:1 d:2 | telomeres: TOP BOTTOM |
| Chr6_Lvf | 74038366 | 553,131,716 | 51.58% | B:548 C:548 F:0 D:0 d:0 | telomeres: TOP |
| Chr7_Lvf | 63982733 | 617,114,449 | 57.54% | B:728 C:727 F:0 D:1 d:1 | telomeres: TOP |
| Chr8_Lvf | 55732288 | 672,846,737 | 62.74% | B:404 C:401 F:0 D:2 d:3 | telomeres: TOP |
| Chr9_Lvf | 39130240 | 711,976,977 | 66.39% | B:374 C:373 F:1 D:0 d:0 |  |
| Chr10_Lvf | 36877371 | 748,854,348 | 69.82% | B:412 C:409 F:1 D:1 d:2 | telomeres: BOTTOM |
| Chr11_Lvf | 30701327 | 779,555,675 | 72.69% | B:420 C:420 F:0 D:0 d:0 |  |
| Chr12_Lvf | 25824471 | 805,380,146 | 75.10% | B:355 C:355 F:0 D:0 d:0 | telomeres: TOP BOTTOM_near BOTTOM |
| Chr13_Lvf | 21584164 | 826,964,310 | 77.11% | B:258 C:258 F:0 D:0 d:0 | telomeres: TOP |
| Chr14_Lvf | 21327804 | 848,292,114 | 79.10% | B:303 C:303 F:0 D:0 d:0 | telomeres: TOP |
| Chr15_Lvf | 20799835 | 869,091,949 | 81.04% | B:290 C:290 F:0 D:0 d:0 | telomeres: TOP |
| Chr16_Lvf | 20111806 | 889,203,755 | 82.91% | B:243 C:242 F:0 D:1 d:1 | telomeres: TOP TOP_near |
| Chr17_Lvf | 18644349 | 907,848,104 | 84.65% | B:251 C:251 F:0 D:0 d:0 | telomeres: TOP BOTTOM |
| Chr18_Lvf | 16541591 | 924,389,695 | 86.19% | B:318 C:316 F:0 D:1 d:2 | telomeres: TOP BOTTOM |
| Chr19_Lvf | 15567225 | 939,956,920 | 87.64% | B:262 C:262 F:0 D:0 d:0 | telomeres: TOP BOTTOM |
| Chr20_Lvf | 15002696 | 954,959,616 | 89.04% | B:274 C:274 F:0 D:0 d:0 | telomeres: TOP |
| Chr21_Lvf | 12777097 | 967,736,713 | 90.23% | B:229 C:229 F:0 D:0 d:0 | telomeres: TOP TOP_near MIDDLE MIDDLE |
| Chr22_Lvf | 12573319 | 980,310,032 | 91.41% | B:223 C:222 F:1 D:0 d:0 | telomeres: MIDDLE |
| Chr23_Lvf | 11756239 | 992,066,271 | 92.50% | B:248 C:248 F:0 D:0 d:0 | telomeres: TOP |
| Chr24_Lvf | 8003886 | 1,000,070,157 | 93.25% | B:188 C:188 F:0 D:0 d:0 | telomeres: TOP BOTTOM |
| Chr25_Lvf | 7744152 | 1,007,814,309 | 93.97% | B:139 C:139 F:0 D:0 d:0 | telomeres: TOP BOTTOM |
| Chr26_Lvf | 7268963 | 1,015,083,272 | 94.65% | B:134 C:134 F:0 D:0 d:0 | telomeres: BOTTOM |
| Chr27_Lvf | 6803409 | 1,021,886,681 | 95.28% | B:154 C:153 F:0 D:1 d:1 | telomeres: TOP |
| Chr28_Lvf | 6444326 | 1,028,331,007 | 95.88% | B:170 C:170 F:0 D:0 d:0 | telomeres: TOP |
| Chr29_Lvf | 6179786 | 1,034,510,793 | 96.46% | B:164 C:163 F:0 D:1 d:1 | telomeres: TOP |
| Chr30_Lvf | 4788275 | 1,039,299,068 | 96.91% | B:85 C:82 F:0 D:2 d:3 | telomeres: TOP BOTTOM |
| Chr31_Lvf | 4683349 | 1,043,982,417 | 97.34% |  | telomeres: MIDDLE BOTTOM |
| Chr32_Lvf | 4644022 | 1,048,626,439 | 97.78% | B:61 C:60 F:0 D:1 d:1 | telomeres: TOP BOTTOM |
| Chr33_Lvf | 2372421 | 1,050,998,860 | 98.00% | B:29 C:29 F:0 D:0 d:0 | telomeres: BOTTOM |
| Chr34_Lvf | 2292617 | 1,053,291,477 | 98.21% |  | telomeres: TOP TOP_near |
| Chr35_Lvf | 1968829 | 1,055,260,306 | 98.39% | B:3 C:0 F:0 D:1 d:3 * | telomeres: MIDDLE BOTTOM |
| Chr36_Lvf | 1929877 | 1,057,190,183 | 98.57% |  | telomeres: TOP |
| Chr37_Lvf | 1896899 | 1,059,087,082 | 98.75% |  | telomeres: TOP |
| Chr38_Lvf | 1688031 | 1,060,775,113 | 98.91% |  |  |
| scaffold_39 | 792425 | 1,061,567,538 | 98.98% |  |  |
| scaffold_40 | 681081 | 1,062,248,619 | 99.05% |  |  |
| scaffold_47 | 617421 | 1,062,866,040 | 99.10% |  |  |
| scaffold_41 | 466723 | 1,063,332,763 | 99.15% |  |  |
| scaffold_42 | 392199 | 1,063,724,962 | 99.18% |  |  |
| scaffold_43 | 352952 | 1,064,077,914 | 99.22% |  | telomeres: TOP |
| scaffold_44 | 352859 | 1,064,430,773 | 99.25% |  |  |
| scaffold_45 | 332306 | 1,064,763,079 | 99.28% |  | telomeres: TOP |
| scaffold_46 | 331746 | 1,065,094,825 | 99.31% | B:1 C:1 F:0 D:0 d:0 | telomeres: TOP |
| scaffold_48 | 274237 | 1,065,369,062 | 99.34% |  |  |
| scaffold_49 | 255847 | 1,065,624,909 | 99.36% |  |  |
| scaffold_50 | 232336 | 1,065,857,245 | 99.38% |  |  |
| scaffold_51 | 222529 | 1,066,079,774 | 99.40% |  |  |
| scaffold_52 | 212371 | 1,066,292,145 | 99.42% |  |  |
| scaffold_53 | 208070 | 1,066,500,215 | 99.44% |  |  |
| scaffold_54 | 195784 | 1,066,695,999 | 99.46% |  | telomeres: TOP |
| scaffold_55 | 183290 | 1,066,879,289 | 99.48% |  | telomeres: TOP |
| scaffold_56 | 178647 | 1,067,057,936 | 99.49% |  |  |
| scaffold_57 | 174567 | 1,067,232,503 | 99.51% |  | telomeres: BOTTOM |
| scaffold_58 | 171796 | 1,067,404,299 | 99.53% |  |  |
| scaffold_59 | 147170 | 1,067,551,469 | 99.54% |  | telomeres: TOP |
| scaffold_60 | 140188 | 1,067,691,657 | 99.55% |  |  |
| scaffold_61 | 137719 | 1,067,829,376 | 99.57% |  |  |
| scaffold_62 | 134803 | 1,067,964,179 | 99.58% |  |  |
| scaffold_63 | 131517 | 1,068,095,696 | 99.59% |  |  |
| scaffold_64 | 127349 | 1,068,223,045 | 99.60% | B:1 C:1 F:0 D:0 d:0 |  |
| scaffold_65 | 126107 | 1,068,349,152 | 99.61% |  |  |
| scaffold_66 | 110870 | 1,068,460,022 | 99.63% |  |  |
| scaffold_67 | 109590 | 1,068,569,612 | 99.64% |  |  |
| scaffold_68 | 105808 | 1,068,675,420 | 99.65% |  |  |
| scaffold_69 | 101436 | 1,068,776,856 | 99.65% |  |  |
| scaffold_70 | 98449 | 1,068,875,305 | 99.66% |  |  |
| scaffold_71 | 91546 | 1,068,966,851 | 99.67% |  |  |
| scaffold_72 | 81735 | 1,069,048,586 | 99.68% |  |  |
| scaffold_74 | 81294 | 1,069,129,880 | 99.69% |  |  |
| scaffold_73 | 81234 | 1,069,211,114 | 99.70% |  |  |
| scaffold_75 | 81173 | 1,069,292,287 | 99.70% |  |  |
| scaffold_76 | 81135 | 1,069,373,422 | 99.71% |  |  |
| scaffold_77 | 78901 | 1,069,452,323 | 99.72% |  |  |
| scaffold_78 | 78067 | 1,069,530,390 | 99.73% |  |  |
| scaffold_79 | 72673 | 1,069,603,063 | 99.73% |  |  |
| scaffold_80 | 72651 | 1,069,675,714 | 99.74% |  |  |
| scaffold_81 | 72397 | 1,069,748,111 | 99.75% |  |  |
| scaffold_82 | 69919 | 1,069,818,030 | 99.75% |  |  |
| scaffold_83 | 68207 | 1,069,886,237 | 99.76% |  |  |
| scaffold_84 | 68123 | 1,069,954,360 | 99.76% |  |  |
| scaffold_85 | 66988 | 1,070,021,348 | 99.77% |  |  |
| scaffold_86 | 66445 | 1,070,087,793 | 99.78% |  |  |
| scaffold_87 | 65391 | 1,070,153,184 | 99.78% |  |  |
| scaffold_88 | 65280 | 1,070,218,464 | 99.79% |  |  |
| scaffold_89 | 64227 | 1,070,282,691 | 99.80% |  |  |
| scaffold_90 | 62549 | 1,070,345,240 | 99.80% |  |  |
| scaffold_91 | 61490 | 1,070,406,730 | 99.81% |  |  |
| scaffold_92 | 60214 | 1,070,466,944 | 99.81% |  |  |
| scaffold_93 | 57685 | 1,070,524,629 | 99.82% |  |  |
| scaffold_94 | 55126 | 1,070,579,755 | 99.82% |  |  |
| scaffold_95 | 54002 | 1,070,633,757 | 99.83% |  |  |
| scaffold_96 | 53407 | 1,070,687,164 | 99.83% |  |  |
| scaffold_97 | 53309 | 1,070,740,473 | 99.84% |  |  |
| scaffold_98 | 53113 | 1,070,793,586 | 99.84% |  |  |
| scaffold_99 | 51578 | 1,070,845,164 | 99.85% |  |  |
| scaffold_100 | 51029 | 1,070,896,193 | 99.85% |  |  |
| scaffold_101 | 49169 | 1,070,945,362 | 99.86% |  |  |
| scaffold_102 | 47514 | 1,070,992,876 | 99.86% |  |  |
| scaffold_103 | 47339 | 1,071,040,215 | 99.87% |  |  |
| scaffold_104 | 46409 | 1,071,086,624 | 99.87% |  |  |
| scaffold_105 | 45961 | 1,071,132,585 | 99.87% |  |  |
| scaffold_106 | 44915 | 1,071,177,500 | 99.88% |  |  |
| scaffold_107 | 43785 | 1,071,221,285 | 99.88% |  |  |
| scaffold_108 | 42595 | 1,071,263,880 | 99.89% |  |  |
| scaffold_109 | 42074 | 1,071,305,954 | 99.89% |  |  |
| scaffold_110 | 39410 | 1,071,345,364 | 99.89% |  |  |
| scaffold_111 | 38733 | 1,071,384,097 | 99.90% |  |  |
| scaffold_112 | 37418 | 1,071,421,515 | 99.90% |  |  |
| scaffold_113 | 36837 | 1,071,458,352 | 99.90% |  |  |
| scaffold_114 | 36251 | 1,071,494,603 | 99.91% |  |  |
| scaffold_115 | 35676 | 1,071,530,279 | 99.91% |  |  |
| scaffold_116 | 34611 | 1,071,564,890 | 99.91% |  |  |
| scaffold_117 | 34461 | 1,071,599,351 | 99.92% |  |  |
| scaffold_118 | 33737 | 1,071,633,088 | 99.92% |  |  |
| scaffold_119 | 33636 | 1,071,666,724 | 99.92% |  |  |
| scaffold_120 | 33209 | 1,071,699,933 | 99.93% |  |  |
| scaffold_121 | 32981 | 1,071,732,914 | 99.93% |  |  |
| scaffold_122 | 31952 | 1,071,764,866 | 99.93% |  |  |
| scaffold_123 | 31535 | 1,071,796,401 | 99.94% |  |  |
| scaffold_124 | 30636 | 1,071,827,037 | 99.94% |  | telomeres: TOP |
| scaffold_125 | 30585 | 1,071,857,622 | 99.94% |  |  |
| scaffold_126 | 30321 | 1,071,887,943 | 99.94% |  |  |
| scaffold_127 | 30188 | 1,071,918,131 | 99.95% |  |  |
| scaffold_128 | 29982 | 1,071,948,113 | 99.95% |  |  |
| scaffold_129 | 29297 | 1,071,977,410 | 99.95% |  | telomeres: TOP_near |
| scaffold_130 | 29174 | 1,072,006,584 | 99.96% |  |  |
| scaffold_131 | 28482 | 1,072,035,066 | 99.96% |  |  |
| scaffold_132 | 27437 | 1,072,062,503 | 99.96% |  |  |
| scaffold_133 | 26543 | 1,072,089,046 | 99.96% |  |  |
| scaffold_134 | 25991 | 1,072,115,037 | 99.97% |  |  |
| scaffold_135 | 25876 | 1,072,140,913 | 99.97% |  |  |
| scaffold_136 | 24322 | 1,072,165,235 | 99.97% |  |  |
| scaffold_137 | 23071 | 1,072,188,306 | 99.97% |  | telomeres: TOP_near |
| scaffold_138 | 22816 | 1,072,211,122 | 99.97% |  | telomeres: TOP |
| scaffold_139 | 22711 | 1,072,233,833 | 99.98% |  |  |
| scaffold_140 | 21735 | 1,072,255,568 | 99.98% |  |  |
| scaffold_141 | 20752 | 1,072,276,320 | 99.98% |  | telomeres: TOP_near |
| scaffold_142 | 20686 | 1,072,297,006 | 99.98% |  |  |
| scaffold_143 | 20552 | 1,072,317,558 | 99.98% |  |  |
| scaffold_144 | 20471 | 1,072,338,029 | 99.99% |  |  |
| scaffold_145 | 20400 | 1,072,358,429 | 99.99% |  |  |
| scaffold_146 | 19697 | 1,072,378,126 | 99.99% |  |  |
| scaffold_147 | 18830 | 1,072,396,956 | 99.99% |  |  |
| scaffold_148 | 18614 | 1,072,415,570 | 99.99% |  |  |
| scaffold_149 | 18076 | 1,072,433,646 | 100% |  |  |
| scaffold_150 | 17734 | 1,072,451,380 | 100% |  | telomeres: TOP_near |
| scaffold_151 | 15505 | 1,072,466,885 | 100% |  | telomeres: TOP_near |
| scaffold_152 | 12661 | 1,072,479,546 | 100% |  |  |

**Candidate Annotation summary stats:**

30101 Genes

13.26% Percentage of Genome

142232926 Total Gene Length

1072479546 Assembly Length

31748 mRNA

5119.83 Mean Length

171634 Longest

201 Shortest

162544457 Total mRNA Length

161303 Exons

5.08 Mean per mRNA

199.33 Mean Length

1012.74 Mean Length per mRNA

13482 Longest Exon

3 Shortest Exon

32152359 Total Exon Length

5056 15.93% Single Exon mRNA

129555 Introns

4.08 Mean per mRNA

993.75 Mean Length

4055.21 Mean Length per mRNA

26550 Longest Intron

41 Shortest Intron

128744773 Total Intron Length

15.16% mRNA

3.00% Exons

12.00% Introns

1072479546 Assembly Length

16468 Genes with gene names

17902 mRNA with gene names

19303 mRNA with gene descriptions

**Candidate Annotation associated files:**

bPyrNan1.0.basic_gff_stats.txt: Contains basic statistics regarding the annotation.

bPyrNan1.0.codingseq.fna: is a fasta file containing nucleotide sequence for each annotated coding sequence.

bPyrNan1.0.faa: is a fasta file containing amino acid sequence for each annotated coding sequence.

bPyrNan1.0.gff: is a general feature format of annotations for the genome.

Table S3. Statistics of nine assembled genomes from birds of the family Tyranidae, including the genome of *Pyrocephalus nanus*.

| Species | Date | Genome size | # Scaffold | Scaffold N50 | # Contigs | Contig N50 | Assembly level | Sequence platform |
| --- | --- | --- | --- | --- | --- | --- | --- | --- |
| *Empidonax traillii* | 3-Apr-2018 | 1.1 Gb | 7791 | 895.1 kb | 45,995 | 86.6 kb | Scaffold | Illumina HiSeq |
| *Myiozetetes cayanensis* | 10-Mar-2022 | 1.1 GB | 1692 | 63.7 Mb | 1,873 | 13 Mb | Scaffold | Oxford nanopore; Illumina |
| *Tyrannus savana* | 10-Jul-2020 | 1.1 GB | 35393 | 436.2 kb | 70,134 | 72.3 kb | Scaffold | Illumina HiSeq |
| *Neopipo cinnamomea* | 10-Jul-2020 | 1 GB | 11198 | 486 kb | 61,449 | 43.1 kb | Scaffold | Illumina HiSeq |
| *Tyrannus tyrannus* | 9-Dec-2022 | 1.1 GB | 43947 | 63.1 Mb | 114,348 | 22.5 kb | Scaffold | Illumina |
| *Pitangus sulphuratus* | 20-Mar-2023 | 1 GB | 11553 | 829.5 kb | 116,115 | 15.7 kb | Scaffold | Illumina MiSeq; Illumina HiSeq |
| *Empidonax alnorum* | 2-Jun-2022 | 1.1 GB | 15260 | 7.7 Mb | 31,625 | 165.8 kb | Scaffold | Illumina HiSeq |
| *Mionectes macconnelli* | 10-Jul-2020 | 1 GB | 4545 | 1.6 Mb | 33,645 | 72.8 kb | Scaffold | Illumina HiSeq |
| *Pyrocephalus nanus* | 16-Nov-2023 | 1.07 GB | 152 | 74.0 Mb | 267 | 17.8 Mb | Chromosome  & scaffold | PacBio Hifi, Illumina HiC |

**References**

Allio R, et al. 2020. MitoFinder: Efficient automated large‐scale extraction of mitogenomic data in target enrichment phylogenomics. Mol Ecol Resour. 20(4):892-905.

Bernt M, et al. 2013. MITOS: improved de novo metazoan mitochondrial genome annotation. Mol Phylogenet Evol. 69(2):313-319.

Katoh K, Standley DM. 2013. MAFFT multiple sequence alignment software version 7: improvements in performance and usability. Mol Biol Evol. 30(4):772-780.

Fig S1. Complete mitochondrial genome was 17151 nucleotides in length as derived from a total of 167 corrected HiFi reads that mapped to the mitogenome with 13 protein coding genes, 22 tRNAs, 2 rRNAs, and 2 control regions, one complete, the other designated as a remnant CR.
