# Supplementary figures and images for: Genome Assembly of Pyrocephalus nanus: A Step Toward the Genetic Conservation of the Endangered Little Vermilion Flycatcher of the Galapagos Islands
Source: Genome Biol Evol. 2024 Apr 23;16(5):evae083. doi: 10.1093/gbe/evae083 (PMC11077314; doi:10.1093/gbe/evae083)

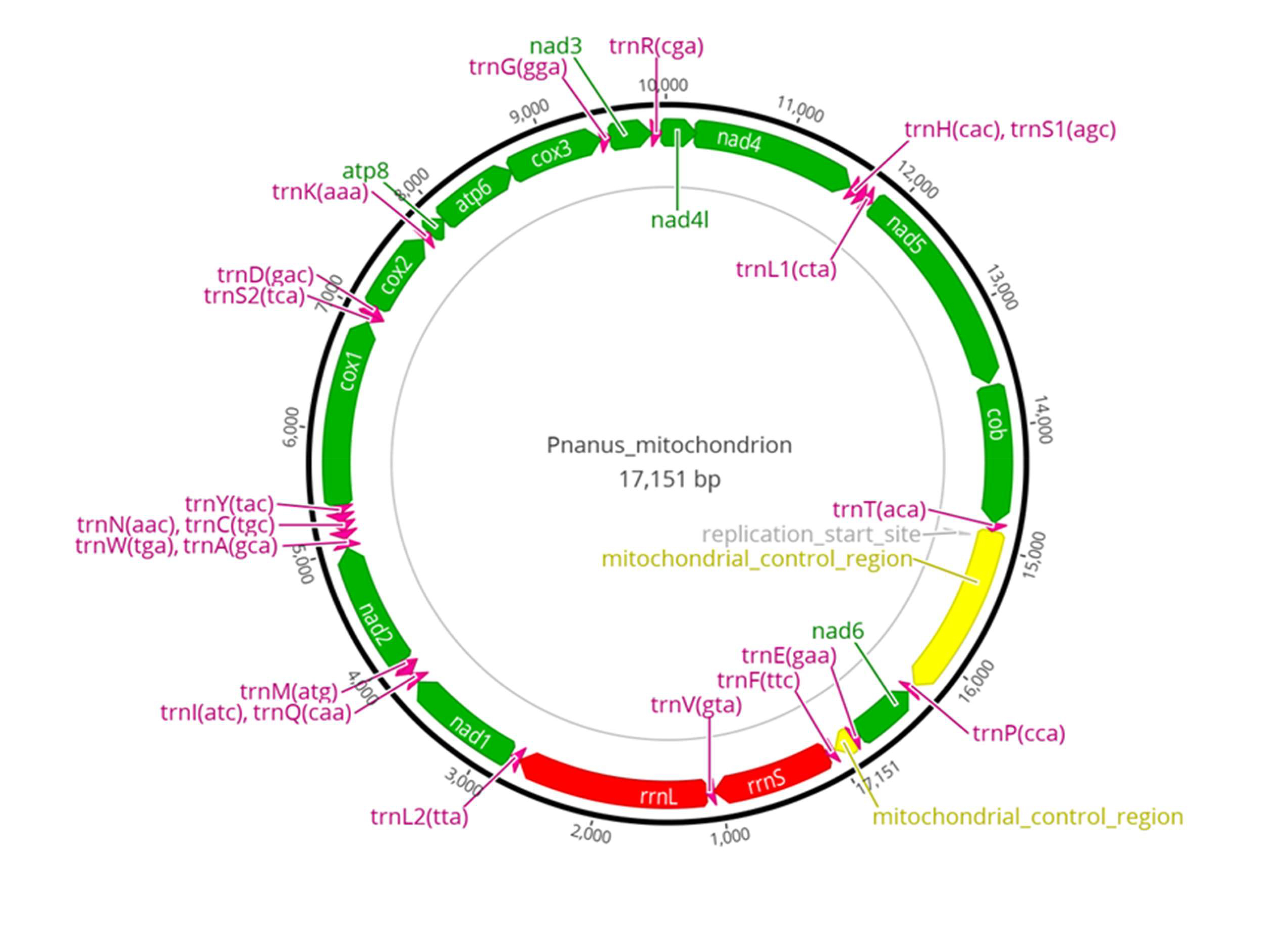

Supplement: evae083_Supplementary_Data [file evae083_supplementary_data.zip › Fig_S1_Supplemental_Mat_LVF_genome_2_April_2024_Final.jpg]
